# Cooperative action of SP-A and its trimeric recombinant fragment with polymyxins against Gram-negative respiratory bacteria

**DOI:** 10.3389/fimmu.2022.927017

**Published:** 2022-09-07

**Authors:** Juan Manuel Coya, Víctor Fraile-Ágreda, Lidia de Tapia, Belén García-Fojeda, Alejandra Sáenz, José A. Bengoechea, Nina Kronqvist, Jan Johansson, Cristina Casals

**Affiliations:** ^1^ Department of Biochemistry and Molecular Biology, Complutense University of Madrid, Madrid, Spain; ^2^ Wellcome-Wolfson Institute for Experimental Medicine, Queen’s University Belfast, Belfast, United Kingdom; ^3^ Department of Biosciences and Nutrition, Neo, Karolinska Institutet, Huddinge, Sweden

**Keywords:** collectin SP-A, recombinant trimeric fragment, multidrug-resistant bacteria, microbial infection, lung, polymyxin B, PMB nonapeptide, synergy

## Abstract

The exploration of therapies combining antimicrobial lung proteins and conventional antibiotics is important due to the growing problem of multidrug-resistant bacteria. The aim of this study was to investigate whether human SP-A and a recombinant trimeric fragment (rfhSP-A) have cooperative antimicrobial activity with antibiotics against pathogenic Gram-negative bacteria. We found that SP-A bound the cationic peptide polymyxin B (PMB) with an apparent dissociation constant (*K*_D_) of 0.32 ± 0.04 µM. SP-A showed synergistic microbicidal activity with polymyxin B and E, but not with other antibiotics, against three SP-A-resistant pathogenic bacteria: *Klebsiella pneumoniae*, non-typable *Haemophilus influenzae* (NTHi), and *Pseudomonas aeruginosa*. SP-A was not able to bind to *K. pneumoniae, NTHi*, or to mutant strains thereof expressing long-chain lipopolysaccharides (or lipooligosaccharides) and/or polysaccharide capsules. In the presence of PMB, SP-A induced the formation of SP-A/PMB aggregates that enhance PMB-induced bacterial membrane permeabilization. Furthermore, SP-A bound to a molecular derivative of PMB lacking the acyl chain (PMBN) with a *K*_D_ of 0.26 ± 0.02 μM, forming SP-A/PMBN aggregates. PMBN has no bactericidal activity but can bind to the outer membrane of Gram-negative bacteria. Surprisingly, SP-A and PMBN showed synergistic bactericidal activity against Gram-negative bacteria. Unlike native supratrimeric SP-A, the trimeric rfhSP-A fragment had small but significant direct bactericidal activity against *K. pneumoniae*, NTHi, and *P. aeruginosa*. rfhSP-A did not bind to PMB under physiological conditions but acted additively with PMB and other antibiotics against these pathogenic bacteria. In summary, our results significantly improve our understanding of the antimicrobial actions of SP-A and its synergistic action with PMB. A peptide based on SP-A may aid the therapeutic use of PMB, a relatively cytotoxic antibiotic that is currently being reintroduced into clinics due to the global problem of antibiotic resistance.

## Introduction

Gram-negative respiratory infections are a persistent and pervasive public health problem (1, 2). This problem has been generated mainly by the uncontrolled expansion of respiratory pathogens resistant to multiple families of antibiotics (3, 4), such as *Klebsiella pneumoniae, Pseudomonas aeruginosa*, and *Haemophilus influenzae*, some of which are listed as ESKAPE pathogens (5, 6). The emergence of antimicrobial resistance in respiratory pathogens limits current antimicrobial treatments, leading to prolonged illness, higher health care expenditures, and an increase in mortality and morbidity rates.

Some of the most well studied virulence factors contributing to the pathogenicity and resistance of Gram-negative bacteria are the polysaccharide capsule (CPS) and lipopolysaccharide (LPS). CPS acts as an external barrier that protects bacteria and mediates the interaction with their environment. LPS is composed of a highly conserved and amphipathic part called lipid A, an oligosaccharide core, and a variable domain of repeated units of oligosaccharide called O-antigen (7). The O-antigen is much longer than the core oligosaccharide and contains the hydrophilic domain of LPS. Wild-type bacteria species with O-chains are termed ‘‘smooth,’’ and hence their LPS are called smooth LPS (S-LPS). Mutants that produce LPS lacking O-specific chains are termed ‘‘rough’’ (R), and their LPS are designated Ra, Rb, Rc, Rd, and Re in order of decreasing core length (7). Both LPS and CPS cause the impermeabilization of bacteria to hydrophobic antibiotics and prevent bacterial recognition by the host’s immune system (8, 9). These virulence factors are involved in bacterial resistance to antimicrobial factors, including antimicrobial proteins and peptides (AMPs) (10, 11).

Currently, clinical research has focused on development of new alternative therapies for replacing and/or supporting classic antimicrobial treatments to combat antibiotic resistance. In this context, proteins and peptides of the innate immune system have attracted huge clinical interest due to their ability to act against these multidrug-resistant pathogens and their reduced ability to induce new bacterial resistance compared to conventional antibiotics (12–14). In addition, they show other characteristics, including broad-spectrum activity, endotoxin neutralization, and potential synergy with antibiotics (15, 16).

Among human lung defense AMPs, surfactant protein A (SP-A) is an oligomeric extracellular protein secreted into the airway mucosa where it recognizes a great variety of immune and non-immune ligands (17–19). SP-A binds to surfactant membranes but also to membrane receptors present in epithelial and immune cells, modifying their response to pathogens or other stimuli. SP-A also recognizes pathogen-associated molecular patterns in some microorganisms, such as the fungi *Pneumocystis carinii* and *Aspergillus fumigatus* (20), the Gram-positive bacteria *Staphylococcus aureus* (21) and *Streptococcus pneumoniae* (22), respiratory syncytial virus (23), and influenza (24). By binding to these pathogens, SP-A facilitates their clearance by macrophages or recruited neutrophils (17, 19). SP-A-deficient mice show decreased microbe clearance and increased tissue markers of inflammation in the lung after infection with group B *Streptococcus*, *Klebsiella pneumoniae, Pseudomonas aeruginosa*, capsulated *Haemophilus influenzae*, respiratory syncytial virus, adenovirus, and influenza virus (25, 26). Although it has been suggested that SP-A has potent direct antibacterial activity *in vivo*, *in vitro* data supporting direct antimicrobial activity of SP-A are sparse. Respiratory pathogens like *K. pneumoniae*, *Bordetella pertussis*, and *P. aeruginosa* are resistant to SP-A (27–31). It is possible that multiple antimicrobial factors are released during immune responses *in vivo*, and lung antimicrobials could act in combination with SP-A. Consistent with this, we recently discovered synergistic antimicrobial action between SP-A and SP-B^N^, an anionic antimicrobial peptide secreted to the alveolar fluid (32). Interaction between SP-A and SP-B^N^ confers new antimicrobial properties, including the ability to kill respiratory pathogens such as *K. pneumoniae* and *P. aeruginosa* that are resistant to either protein alone (30, 31).

Each SP-A subunit consists of an N-terminal segment containing cysteine residues involved in oligomerization followed by a collagen-like region, an alpha helical coiled neck region, and a globular region with a carbohydrate recognition domain (CRD) (17–19). SP-A is intracellularly assembled in multiples of three subunits *via* its collagen domain. Its supratrimeric assembly has an umbelliform-shaped structure of six trimers like mannose binding protein or C1q (17–19). Supratrimeric oligomerization of SP-A appears to be needed for many of its functions since it facilitates multivalent binding and increases the functional affinity of the globular domain for their ligands (33, 34). However, there are clear advantages of using the smaller recombinant fragments of SP-A, in terms of ease of production and delivery, if they are as effective as native SP-A. In this study we use a recombinant trimeric fragment of human SP-A1 (rfhSP-A), which lacks the N-terminal domain and most of the collagen domain, to evaluate its bactericidal activity alone or in combination with antibiotics. We previously showed that rfhSP-A is highly effective in neutralizing respiratory syncytial virus (23).

We hypothesize that combinations of SP-A, or its recombinant fragments, with antibiotics against Gram-negative bacteria, such as polymyxins, could be an effective antibacterial strategy. Polymyxins are relatively cytotoxic antibiotics that have been reintroduced into clinics as the last-resort therapy for severe MDR infections (35). Polymyxins are pentacationic lipopeptide antibiotics expressed by *Bacilus polymyxia*, which act only against Gram-negative bacteria. They carry five free amino groups in a polycationic peptide ring and a tripeptide side chain with a fatty acid tail (36). The main mechanism of action consists of electrostatic interaction between the negative charge of LPS and the positive charge of the peptide, followed by insertion of the polymyxin molecule into the bacterial membrane. The cyclic lipopeptide cross-links the outer membrane and cytoplasmic bacterial membranes, leading to permeabilization of both membranes and resulting in lysis and cell death (36, 37). However, polymyxins’ mechanism is more complex than their effects in membrane permeabilization because polymyxins bind ribosomes, prevent cell division, and inhibit bacterial respiration (37). Polymyxin derivatives have also acquired great interest with the aim of reducing lipopeptide toxicity and obtaining efficient antimicrobial therapies (38). For example, polymyxin B nonapeptide (PMBN), a PMB derivative that lacks the fatty acid tail and the N-terminal diaminobutyryl residue, retains polymyxin B’s ability to bind to LPS and disturb the outer membrane of Gram-negative bacteria. However, PMBN has no bactericidal activity on its own and its use depends on efficient combination with other antimicrobial factors (38, 39).

The objectives of this article are to investigate i) the bactericidal activity of human SP-A and rfhSP-A, produced in a novel way using the spider silk derived solubility tag NT* (40, 41), against clinically relevant respiratory pathogens and isogenic mutants, and ii) the synergistic or additive antimicrobial activity of human SP-A and rfhSP-A with antibiotics such as polymyxins against these respiratory pathogens. Since SP-A is the most abundant protein in the alveolar space, comprehensive understanding of protein-polymyxin interactions would facilitate the development of new therapies against respiratory infections by Gram-negative bacteria, which induce airway attacks in patients who are elderly or who suffer from chronic obstructive pulmonary disease (COPD), cystic fibrosis, or asthma.

## Material and methods

### Materials

The antibiotics azithromycin (AZI), ciprofloxacin (CIPRO), tetracycline (TC), polymyxin B (PMB), colistin (PME) and PMB nonapeptide (PMBN) were obtained from Sigma-Aldrich (St. Louis, MO, USA). Antibiotics were prepared according to the manufacturer’s instructions; the stocks of antibiotics were diluted in the corresponding dilution media and were used on the same day of dilution. The β-Nicotinamide adenine dinucleotide hydrate (β-NAD) and hemin were obtained from Sigma-Aldrich. Sytox Green, propidium iodide and 1,6-diphenyl-1,3,5-hexatriene (DPH) fluorescent dyes were from Molecular Probes (Eugene, OR, USA). The fluorescent dye 5 (6)-carboxyfluorescein diacetate *N*-succinimidyl ester (CFSE) was obtained from ThermoFisher Scientific (Waltham, MA, USA). Chocolate agar plates for the growth of nontypeable *Haemophilus influenzae* strains were from bioMérieux (Marcy l’Etoile, France). Rough lipopolysaccharide (Re 595, Re-LPS) from *Salmonella enterica* serotype Minnesota was obtained from Sigma-Aldrich. Dipalmitoylphosphatidylcholine (DPPC) was obtained from Avanti Polar Lipids (Birmingham, AL, USA). All other reagents were obtained from Sigma-Aldrich.

### Bacterial strains and growth conditions

*K. pneumoniae* 52145 (serotype K2:O1), nontypeable *Haemophilus influenzae* strain 375 (NTHi), and *P. aeruginosa* (PAO1) are clinical isolates, as previously described (42–45). The chemical structure of *K. pneumoniae* and PAO1 capsules has been reported (46, 47). **Table 1** summarized *K. pneumoniae* 52145 wildtype (WT) and isogenic mutants with or without capsule and expressing a range of different LPS phenotypes used in this study. **Table 1** also shows NTHi 375 and mutant strains with defects in outer membranes (OM) used in this study. They were generated as previously described (47–49). *P. aeruginosa*, *K. pneumoniae* K2, and its mutant strains were grown in Luria–Bertani (LB) broth at 37°C with continuous shaking to the exponential phase. Frozen stocks of NTHi strains were thawed and then grown on chocolate agar plates during 18 h at 37°C in a humidified 5% CO_2_ atmosphere. Then, NTHi were grown to the exponential phase on brain heart infusion broth (BHI) supplemented with 10 µg/ml hemin and 10 µg/ml β-NAD (sBHI) with continuous shaking at 37°C in a humidified 5% CO_2_ atmosphere. Exponential-phase bacteria were then harvested, resuspended in PBS, and adjusted to the desired final concentration, as described in (30, 31).

**Table 1:** Bacterial strains used in this study.

Bacterial strains	Nomenclature in the text	LPS Serotype	CPS	Ref
***Klebsiella***				
*K. pneumoniae* 52145	wt Kp	S-LPS	CPS (K2)	(42, 48)
52145-Δ*wca*_K2_	wt Kp-CPS	S-LPS	–	(10)
52145-Δ*wabK*	Rc Kp+CPS	Rc-LPS	CPS (K2)	(47)
52145-Δ*wca*_K2_-Δ*wabK*	Rc Kp-CPS	Rc-LPS	–	(48)
52145-Δ*waaC*	Re Kp-CPS	Re-LPS	–	(48)
***Haemophilus***				
*Nontypeable H. influenzae* 375	wt-NTHi	S-LOS	–	(44, 49)
375 Δ*lgtF-*Δ*lpsA*	Rc-NTHi	Rc-LOS	–	(49)
375 Δ*opsX*	Re-NTHi	Re-LOS	–	(49)
***Pseudomonas***				
*P. aeruginosa*	PAO1	S-LPS	CPS (Alginate)	(43)

S-, smooth; R(c, e)-, rough; LOS, lipooligosaccharide; CPS, capsule.

### Isolation of human SP-A

Surfactant protein A was isolated from bronchoalveolar lavage of patients with alveolar proteinosis using a sequential n-butanol and octylglucoside extraction (30, 31, 33, 34, 50, 51). The purity of SP-A was evaluated by one-dimensional SDS-PAGE in 12% acrylamide under reducing conditions and mass spectrometry. SP-A structure was analyzed by tryptophan fluorescence and circular dichroism as in (30, 31, 33, 34, 50, 51). SP-A hydrodynamic diameter was determined by dynamic light scattering as in (30, 51). The degree of SP-A oligomerization was assessed by electrophoresis under nondenaturing conditions, electron microscopy, and analytical ultracentrifugation as reported elsewhere (33, 34). SP-A consisted of supratrimeric oligomers of at least 18 subunits (molecular mass, 650 kDa). Each subunit had an apparent molecular mass of 36,000 Da. Endotoxin content of isolated human SP-A was about 300 pg endotoxin/mg SP-A, as determined by Limulus amebocyte lysate assay (Bio-Whittaker, Walkersville, MD, USA).

### Expression and purification of rfhSP-A

The recombinant trimeric fragment of human SP-A (rfhSP-A) (molecular mass, 57 kDa), including the globular carbohydrate recognition domain (CRD), neck, and 8 x Gly-Xaa-Yaa repeats of the collagen stalk, was previously expressed in fusion with the wild-type NT solubility tag and purified by refolding (23). In the present study, rfhSP-A was sub-cloned into a pT7 expression vector containing the NT* tag N-terminally of the rfhSP-A (40, 41). A His_6_-tag was included in the N-terminal of NT* to allow efficient purification. The cleavage site for coxsackievirus 3C protease was added between NT* and rfhSP-A to allow removal of the tag after purification. BL21 (DE3) *Escherichia coli* containing the plasmid encoding NT*-rfhSP-A were grown over night at 37°C in LB media containing 70 mg/L kanamycin. 10 mL culture was used to inoculate 1 L of LB medium with kanamycin and the cells were grown at 30°C to OD_600_ ~ 0.9. Isopropyl β-D-1-thiogalactopyranoside (IPTG) was added to a concentration of 0.5 mM and protein was expressed for 20 h at 20°C. Cells from 1 L culture were harvested by centrifugation at 4000 xg for 20 min and the pellet was resuspended to 60 mL in 20 mM Tris-HCl, 2 M urea, pH 8. The cell solution was sonicated (Sonics VC505 ultrasonic processor, converter model CV334, standard probe 13 mm) at 80% amplitude, 1 sec pulses, for a total of 2 min 40 sec. After lysis, a clear supernatant was obtained by centrifugation at 27,000 x g, 4°C for 30 min. The supernatant was loaded to a Ni-sepharose column (GE Healthcare) equilibrated with 20 mM Tris-HCl, 2 M urea, pH 8. The bound protein was washed with Tris buffer containing decreasing concentrations of urea (2 M, 1 M, 0.5 M and no urea) until the A_280_ baseline was reached. The protein was eluted with 20 mM Tris-HCl, 300 mM imidazole, pH 8 and imidazole was removed by over-night dialysis using a Spectra/Por^®^ membrane with a 6-8 kDa molecular weight cut-off placed in 5 L of 20 mM Tris-HCl, pH 8 at 4°C. The fusion protein was cleaved at 4°C overnight using 1:10 (w/w) 3C protease in the presence of 1 mM DTT. An over-night dialysis was performed as described above to remove DTT, and rfhSP-A was purified by reapplication to an IMAC column to remove His-tagged NT* and 3C protease. The protein was concentrated to 1.4 mg/mL using a Vivaspin 20 centrifugal tube with a 5 kDa molecular weight cut-off (GE Healthcare). rfhSP-A identity was evaluated by one-dimensional SDS-PAGE. LPS contamination was removed by addition of polymyxin B-agarose to the rfhSP-A sample in 5 mM Tris, 150 mM NaCl, pH 7.4, at 1:5 (vol/vol). OGP (30 mM) was also added to the suspension. The sample was incubated for 30 min at room temperature in a rotator shaker and centrifuged at 500 g for 5 min at 4°C. The supernatant was then dialyzed, and the protein was quantified by the Lowry method. Endotoxin content was then determined by Limulus amebocyte lysate assay. Structural characteristics of rfhSP-A were assessed by tryptophan fluorescence and circular dichroism as in (33, 34, 50), and its hydrodynamic size by dynamic light scattering (30, 51).

### Bacterial killing assays

The microbicidal activity of SP-A, rfhSP-A, and/or conventional antibiotics was evaluated by colony counts on plate assays (11, 30, 31). Five microliters of bacterial suspension (10^5^ CFU/ml) were incubated with different concentrations of proteins (SP-A or rfhSP-A), antibiotics, and combinations thereof in 30 μL of 10 mM phosphate buffer, 100 mM NaCl, and 1% tryptic soy broth (*Liquid Testing Medium*, LTM) for 1 h at 37°C. At the end of incubation, bacterial suspensions were plated on LB agar for *K. pneumoniae* and PAO1 strains, or sBHI agar for NTHi strains, and incubated for 18 h at 37°C. Viable bacteria were enumerated by colony count. The results were expressed as a percentage of relative survival in comparison to untreated bacteria. The molar concentration of rfhSP-A used was 6 times higher than that of SP-A, since native SP-A is composed of 6 trimerics units.

Direct bactericidal activity of SP-A was assessed by fluorescence microscopy using *K. pneumoniae* 52145 and mutant strains transformed with pZsGreen-expressing GFP or NTHI strains stained with CFSE (52). Briefly, 100 μl of GFP expressing *K. pneumoniae* suspension, containing 10^8^ CFU/ml, were incubated with or without 100 μg/ml SP-A in LTM for 1 h at 37°C. On the other hand, 100 μl of a CFSE-stained NTHi suspension, containing 10^8^ CFU/ml, were incubated with or without 25 or 100 μg/ml SP-A in Hank’s balanced salt solution (HBSS) (0.137 M NaCl, 5,4 mM KCl, 0.25 mM Na_2_HPO_4_, 0.44 mM KH_2_PO_4_, 1.3 mM CaCl_2_, 1 mM MgSO_4_, 4.2 mM NaHCO_3_) supplemented with 1% tryptic soy broth for 1 h at 37°C in a humidified 5% CO_2_ atmosphere. Polymyxin B was used as a positive killing control. Samples were then stained with 30 μM propidium iodide, a membrane-impermeable fluorescent probe, for 15 min under dark conditions. Bacteria were pelleted, re-suspended in the same buffer, and mounted on glass slides. Living (green) and dead/dying (red) bacteria were visualized by fluorescence microscopy (Leica TCS SP2 Confocal System).

### Binding of SP-A to bacteria

To explore the ability of SP-A to bind to *K. pneumoniae* 52145 (or mutant strains) in the absence or presence of polymyxins (PMB and PMBN), binding assays were performed with biotinylated SP-A, which was prepared as previously described (51). Structure and functional activities of biotinylated SP-A were similar to those of unlabeled SP-A. The binding assay of biotinylated SP-A to bacteria was executed as previously described (30, 31). Briefly, 10^7^ CFU/ml log-phase bacteria in 5 mM Tris-HCl buffer containing 150 mM NaCl and 2 mM Ca^2+^ were incubated with several concentrations of biotinylated SP-A in the absence or presence of PMB (5 μg/ml), PMBN (5 μg/ml), or human serum albumin (HSA) in 10% FBS-blocked microcentrifuge tubes by gentle orbital rotation for 30 min at room temperature. In all cases, bacteria were pelleted, washed twice, and resuspended in 200 ml carbonate buffer 0.1 M, pH 9.5. Controls were performed in the absence of bacteria to estimate nonspecific binding. Bacteria-associated SP-A was next measured by solid-phase binding as follows. Samples were applied to a 96-well plate MaxiSorp (Nunc, Rochester, NY, USA) and allowed to bind 1 h at 37°C. The plate was next blocked with 5 mM Tris-HCl containing 10% FBS for 1 h at 37°C. After extensive washing with PBS, streptavidin-HRP was added to the wells and incubations were performed for 1 h at room temperature. Biotinylated SP-A was detected by adding 3,3’,5,5’-tetramethylbenzidine liquid substrate. The colorimetric reaction was halted with 4M sulfuric acid, and absorbance read at 450 nm on an ELISA reader (DigiScan, Asys HiTech GmbH, Eugendorf, Austria). Results were expressed as nanograms of SP-A per 10^7^ bacteria.

### Bacterial aggregation assays

Bacteria in exponential phase were re-suspended in HBSS buffer and adjusted to a final concentration of 10^9^ CFU/ml (OD_700_ = 1). For PAO1, *K. pneumoniae* 52145 and mutant strains, either SP-A (25 μg/ml) or rfhSP-A (13 µg/ml) was added to 150 μl of the bacterial suspension and incubated for 30 min at 37°C with 30 seconds of gentle shaking every 5 min. Bacterial aggregation was determined by monitoring changes in absorbance at 700 nm during 2 h at 37°C without shaking, in a spectrophotometer DU-800 (Beckman Coulter, Fullerton, USA). Bacterial aggregation is observed as a decrease in absorbance as bacterial aggregates precipitate out of solution. SP-A-induced bacterial aggregation was also visualized by fluorescence microscopy (Leica TCS SP2 Confocal System) using *K. pneumoniae* 52145 and mutant strains expressing GFP. Bacterial suspensions (10^9^ CFU/ml) in HBSS buffer were incubated with SP-A (25 μg/ml) in FACS tubes in the conditions described above for direct visualization. For NTHi and mutant strains, which exhibit aggregation on their own (52), the process of SP-A-induced bacterial aggregation was measured immediately after addition of SP-A or buffer by measuring changes in light absorbance at 700 nm during 2 h without shaking.

### Intrinsic fluorescence experiments

To assess the binding of human SP-A and its recombinant fragment to PMB or PMBN, the tryptophan fluorescence of rfhSP-A or SP-A was used as in (30, 50, 53, 54) to determine the apparent dissociation constant (*K_D_*) at 25°C for protein-antibiotic complexes. Experiments were carried out in 5x5 quartz cuvettes using an SLM-Aminco AB-2 spectrofluorimeter equipped with a thermostated cuvette holder ( ± 0.1°C; Thermo Spectronic, Waltham, MA, USA). Fluorescence emission spectra of SP-A (15 nM) or rfhSP-A (0.3 μM) in the absence and presence of increasing concentrations of polymyxins were recorded from 305 to 400 nm on excitation at 295 nm at 25°C in 5 mM Tris-HCl buffer (pH 7.4) containing 150 mM NaCl or not. Each titration data point was performed in separated samples, and tryptophan fluorescence emission was monitored 10 min after PMB or PMBN addition. The change in the fluorescence of SP-A (or rfhSP-A) at the emission wavelength maximum was monitored as a function of PMB or PMBN concentration, and the titration data were analyzed by nonlinear least-squares fitting to the Hill equation, as previously reported (30, 53, 54):


(1) $$\Delta F/\Delta F_{max}={\left[L\right]}^{nH}/({\left[L\right]}^{nH}+K_{D})$$

where *ΔF* is the change in fluorescence intensity at 337 nm relative to intensity of free SP-A (for free rfhSP-A, the emission wavelength maximum was 342 nm); *ΔF_max_* is the change in fluorescence intensity at saturating polymyxin concentrations; *K_D_* is the apparent equilibrium dissociation constant; [L] is the molar concentration of free PMB or PMBN; n*_H_* is Hill coefficient.

### Dynamic light scattering

These assays were carried out as in (30, 51, 54–56) to determine if there are changes in the size of the particles after the interaction of SP-A (or rfhSP-A) with the corresponding polymyxin (PMB or PMBN) in solution. Hydrodynamic diameters of PMB, PMBN, SP-A, rfhSP-A, and combinations thereof were measured at 25°C in a Zetasizer Nano S (Malvern Instruments, Malvern, UK) equipped with a 633-nm HeNe laser. Interaction of SP-A or rfhSP-A with polymyxins in solution was measured by addition of different concentrations of polymyxins to 15 nM SP-A (or rfhSP-A) in 5 mM Tris-HCl buffer (pH 7.4), in the absence or presence of 150 mM NaCl. Ten scans were performed for each sample, and experiments were performed in triplicate. The hydrodynamic diameters were calculated using the general purpose and multiple narrow modes algorithms available from the Malvern software for DLS analysis.

### Bacterial membrane permeabilization

The ability of SP-A (or rfhSP-A), PMB, PMBN, and combinations thereof to permeabilize the outer and cytoplasmic bacterial membranes was studied in live bacteria by quantifying the internalization of the impermeant fluorescent Sytox Green, since its fluorescence increases when binding to bacterial DNA (31). For the measurement of Sytox Green influx, the probe (1 μM) was added to 1 ml of bacterial suspension (2x10^7^ CFU/ml) in LTM and the sample was incubated for 15 min in darkness at room temperature. Then, the fluorescence of the Sytox Green/bacterial suspension mixture was monitored for 4 hours in a FLUOstar Omega microplate reader (BMG LabTechnologies, Ortenberg, Germany) at excitation and emission wavelengths of 485 and 520 nm, respectively. PBS was used as a negative control, whereas ethanol (70%) was used as a positive control. Background fluorescence was measured in non-labeled bacteria.

### Bacterial membrane alteration

Exponential phase bacteria (1×10^7^ CFU/mL) were treated with SP-A (100 µg/mL), PMB (1 µg/mL), PMBN (1 µg/mL) and combinations thereof at 37°C for 30 min in PBS buffer as previously described in (31). The suspension was incubated with 20 μM of DPH dissolved in N,N-dimethylformamide for 1h at 37°C in darkness. DPH fluorescence intensity was measured using an SLM-Aminco AB-2 spectrofluorimeter equipped with a thermostated cuvette holder (Thermo Spectronic, Waltham, MA, USA). Quartz cuvettes of 5x5-mm path length were used. Excitation and emission wavelengths: 350 and 450 nm, respectively. Non-labeled bacteria were used as background. All experiments were conducted in triplicate.

### LPS aggregation assays

LPS aggregation induced by SP-A, PMB, PMBN, and combinations thereof was studied as described elsewhere (33, 34, 57) by measuring the change in absorbance at 400 nm in a Beckman DU-800 spectrophotometer. Briefly, the sample and reference cuvettes were first filled with Re-LPS in 5 mM Tris-HCl buffer (pH 7.4), 150 mM NaCl, and 0.2 mM EDTA. After a 10-min equilibration at 37°C, SP-A, PMB, PMBN, or combinations thereof were added to the sample cuvette, and the change in optical density at 400 nm was monitored. Next, Ca^2+^ (2.5 mM) was added to both the sample and reference cuvettes, and the change in absorbance was monitored again. For LPS aggregation assay, final concentrations (58) were 40 μg/ml Re-LPS, 20 μg/ml (30 nM) SP-A, 1.5 μg/ml (1.08 µM) PMB, and 2.5 μg/ml (2.59 µM) PMBN. DPPC vesicle aggregation experiments were also performed as previously reported (33, 34).

### Statistical analysis

Data are presented as means ± SDs. Differences in the means were analyzed by one-way ANOVA followed by the Bonferroni multiple-comparison test. For comparison of two groups, Student t test was used. An α level ≤ 5% (p ≤ 0.05) was considered significant.

## Results

### Direct antimicrobial activity of SP-A against *K. pneumoniae* K2 and isogenic mutants

**Figure 1:**
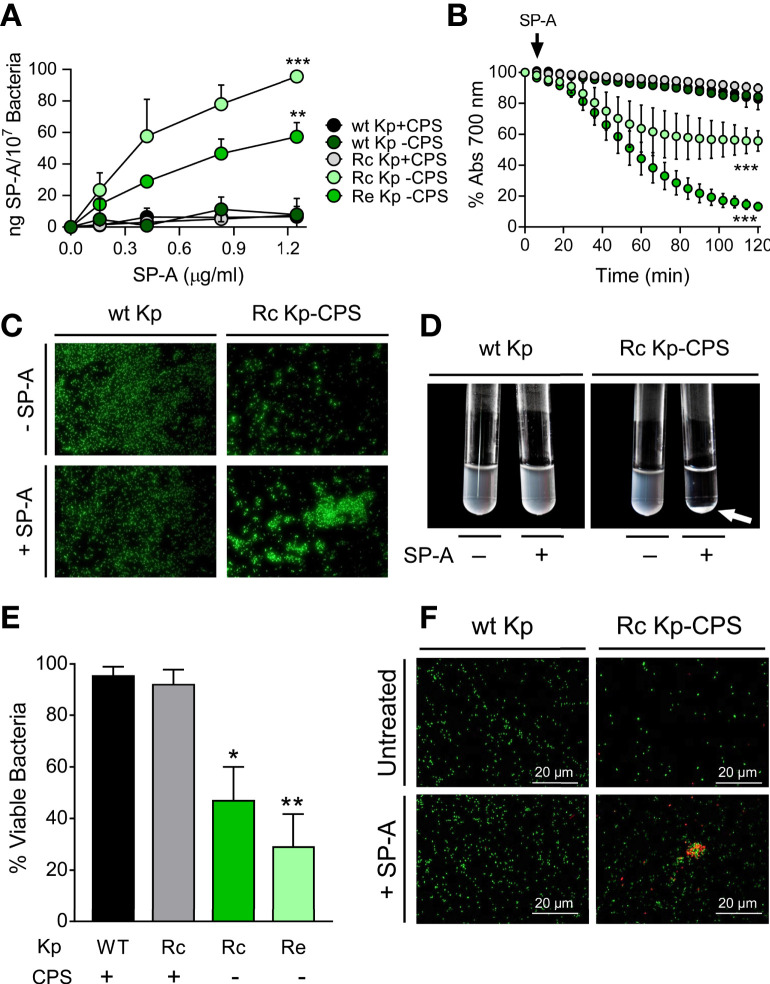
SP-A binds, aggregates, and kills deep rough non-capsulated *K pneumoniae*, but not strains expressing long-chain LPS and/or capsule. **(A)** *K pneumoniae* strains (10^7^ CFU) were incubated with biotinylated SP-A (0 to 1.25 µg/ml), and total *Klebsiella*-associated SP-A was measured by solid-phase binding and expressed as total ng of SP-A/10^7^ bacteria. Data are means ± SD of three independent experiments. A value of ***p < 0.001 was obtained for the one-way ANOVA, followed by the general multiple-comparison Bonferroni test. **(B)** Bacterial aggregation by SP-A (25 μg/ml) was determined by monitoring the changes in absorbance at 700 nm during 2 h at 37°C without shaking. Absorbance of strains incubated in the absence of SP-A corresponds to 100%. Data are means ± SD (n=3, each in triplicate). **(C)** SP-A-induced bacterial aggregation was visualized by fluorescence microscopy using wt and Rc-CPS *K pneumoniae* strains expressing GFP. Images shown are representative of three independent experiments with similar results. Scale bar, 20 μm. **(D)** SP-A-induced bacterial aggregation of the Rc-CPS strain but not of the wt strain was also visualized by the formation of bacterial pellets (white arrow) after 2 h of incubation with 25 μg/ml SP-A. **(E)** The antimicrobial activity of SP-A on *K pneumoniae* strains was determined by incubation of bacteria (10^5^ CFUs/ml) in the absence or presence of SP-A (100 µg/ml) for 1 h at 37°C. Then, bacteria were plated on LB agar for CFU count after 18 h of incubation at 37°C. The results are shown as % viable bacteria (percentage of live colony counts compared with untreated control). Data are means ± SD of three independent experiments with three biological replicates. **(F)** Bacterial killing was also visualized by fluorescence microscopy. Strains expressing GTP (wt Kp and Rc Kp-CPS) were incubated with or without 100 µg/ml SP-A and stained with propidium iodide to assess viability. Images shown are representative of three independent experiments. Scale bar, 20 μm. In **(A)**, **(B)**, and **(E)**, results were statistically analyzed by one-way ANOVA followed by the Bonferroni multiple-comparison test. *p < 0.05, **p < 0.01, and ***p < 0.001 when comparing SP-A-treated with untreated *K pneumoniae* strains.

*K. pneumoniae* is a common nosocomial Gram-negative bacterium that expresses S-LPS and CPS, which can be classified into 77 serotypes; K1 and K2 serotypes are expressed by a significant number of clinical isolates (59). We investigated SP-A antimicrobial activity against *K. pneumoniae* 52145 (serotype K2) wild-type (WT) and isogenic mutants expressing a range of different LPS phenotypes (**Table 1**) by determining its ability to bind, aggregate, and kill bacteria (**Figure 1**). This *K. pneumoniae* strain encodes all the virulence determinants associated with invasive strains (60, 61). SP-A bound to both Rc and Re non-capsulated *K. pneumoniae* strains in a dose-dependent manner, but it did not bind to capsulated WT and Rc strains and the non-capsulated WT strain, as seen by solid-phase binding assay (**Figure 1A**). These results indicate that the K2 capsule and LPS glycoconjugate structures underneath the K2 capsule are not recognized by native SP-A. Consistent with bacterial binding assays, spectrophotometry experiments revealed that SP-A induces aggregation of non-capsulated Rc and Re strains in the presence of calcium, but not bacterial strains to which SP-A does not bind (**Figure 1B**). Bacterial aggregation is observed as a decrease in absorbance as bacterial aggregates precipitate out of solution. SP-A’s ability to aggregate non-capsulated deep rough *K. pneumoniae* strains was confirmed by fluorescence microscopy using strains transformed with pZsGreen-expressing GFP (**Figure 1C**) and bacterial pellet formation (**Figure 1D**). SP-A exhibited direct microbicidal activity against non-capsulated Rc and Re strains, but not against capsulated WT and Rc and non-capsulated WT strains (**Figure 1E**). These results were confirmed using the membrane-impermeable DNA-specific dye propidium iodide. Following incubation with SP-A, the non-capsulated Rc strain, but not the *K. pneumoniae* K2 strain, formed aggregates, and most bacteria were propidium iodide-stained, indicating bacterial cell death (**Figure 1F**). Together, these results indicate that LPS and CPS glycoconjugates of *K. pneumoniae* confer protection from direct antimicrobial activity of native human SP-A.

### Direct antimicrobial activity of SP-A against nontypeable *Haemophilus influenzae* and isogenic mutants.

**Figure 2:**
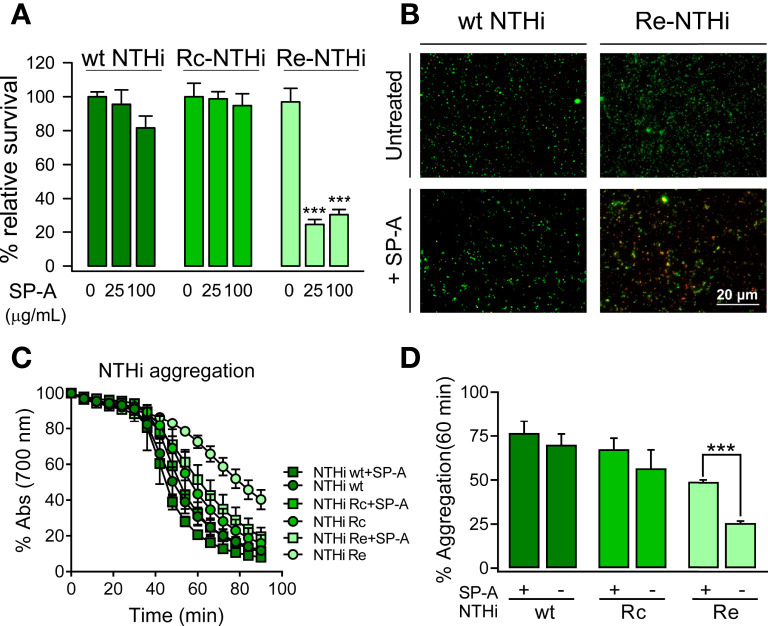
SP-A kills and induces aggregation of deep rough NTHi strains but not of NTHi strains expressing long-chain LOS. **(A)** Bacteria (10^5^ CFUs/ml) were incubated with and without SP-A (25 and 100 µg/ml) and plated on sBHI agar for CFU count. The results are shown as % viable bacteria (percentage of live colony counts compared with untreated control). Data are means ± SD of three independent experiments with three biological replicates. **(B)** Bacterial killing was also visualized by fluorescence microscopy using wt NTHi and Re-NTHi stained with CFSE. Bacteria were incubated with 100 µg/ml SP-A for 1 h and then stained with 30 μM propidium iodide to assess viability. Images shown are representative of three independent experiments. Scale bar, 20 μm. **(C)** SP-A-induced aggregation of NTHi strains was monitored by changes in absorbance at 700 nm during 1.30 h at 37°C without shaking in HBSS buffer. **(D)** Percentage of aggregation at 60 min is shown. Data are means ± SD (n=3, each triplicate). In **(A, D)**, results were statistically analyzed by one-way ANOVA followed by the Bonferroni multiple-comparison test. ***p < 0.001 when the Re-NTHi strain treated with SP-A was compared with that not treated with SP-A.

Nontypeable *H. influenzae* (NTHi) is a human-restricted respiratory pathogen (62) expressing surface glycolipids that lack O-antigens and have been termed lipooligosaccharides (LOS) (49). Since LOS glycoconjugates of NTHi have been shown to be involved in NTHi pathogenicity and antimicrobial resistance (49), we next determined SP-A antibacterial activity against strain NTHi375, clinical isolate from a patient with otitis media, and isogenic mutants with truncated LOS variants (**Table 1**). SP-A exhibited direct microbicidal activity against the deep rough mutant Re-NTHi strain, which conserves only the KDO component of LOS, but did not significantly kill the wild-type strain (with complete LOS) and the Rc-NTHi strain (lacking the outer part of LOS) (**Figure 2A**). These results support the role of LOS glycoconjugates in NTHi resistance to SP-A. The results were confirmed by fluorescence microscopy using wt NTHi and mutant strains stained with CFSE. After SP-A treatment, the deep rough mutant Re-NTHi strain, but not the wild-type strain, were propidium iodide-stained, indicating bacterial death (**Figure 2B**). NTHi and mutant strains have the property to aggregate on their own in the presence of calcium (52). The rate of auto-aggregation of NTHi depends on the complexity of LOS. It decreases progressively with decreasing LOS extension in Rc-NTHi and Re-NTHi strains (**Figure 2C**). The presence of SP-A significantly increases aggregation of the deep rough mutant Re-NTHi strain, but not the wild-type and Rc-NTHi strains (**Figures 2C, D** **)**.

### Interaction of SP-A with antibiotics.

**Figure 3:**
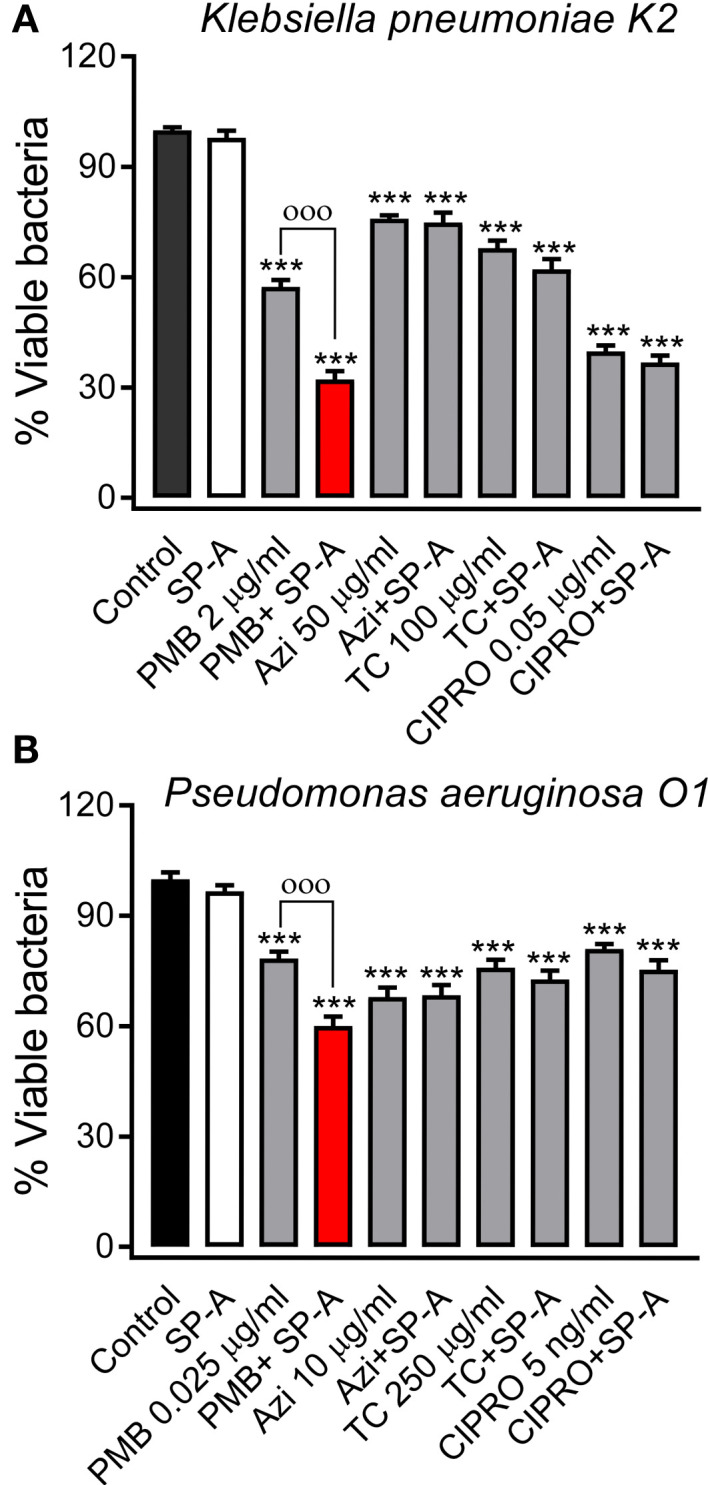
SP-A has synergic antimicrobial activity with polymyxin B (PMB), but not with azithromycin (AZI), tetracycline (TC), and ciprofloxacin (CIPRO), against Gram-negative bacteria. 10^5^ CFU/ml of *K pneumoniae* **(A)** or PAO1 **(B)** were incubated with conventional antibiotics, at the indicated concentrations, in the absence or presence of SP-A (100 μg/ml) in 10 mM phosphate buffer (pH 7.4), 1% TSB, and 100 mM NaCl for 1h at 37°C. Then, the bacteria were plated on LB agar for CFU count after 18 h of incubation at 37°C. The results are shown as % viable bacteria (percentage of live colony counts compared with untreated control). SP-A alone was unable to kill capsulated *K pneumoniae* or PAO1, as previously reported (25). Data are the means ± SD of three independent experiments with at least three biological replicates. A value of p < 0.001 was obtained for the one-way ANOVA, followed by the general multiple-comparison Bonferroni test: ***p < 0.001 when comparing antibiotic treatment vs. the control untreated group; ^ооо^ *p* < 0.001 when comparing SP-A+antibiotic treatment vs. the antibiotic-treated group.

We next evaluated the potential synergy of SP-A with conventional antibiotics against Gram-negative bacteria. To achieve this, we used polymyxin B and E, azithromycin, tetracycline, and ciprofloxacin because they have different mechanisms of action and different targets on Gram-negative bacteria (58). **Figure 3** shows that SP-A had synergistic antimicrobial activity with sub-inhibitory concentrations of polymyxin B (PMB) against *K. pneumoniae* (**Figure 3A**) and *P. aeruginosa* (**Figure 3B**), which are resistant to SP-A. However, SP-A did not show synergy with other antibiotics such as azithromycin, tetracycline and ciprofloxacin. In these experiments, both SP-A and the antibiotics were in solution. When bacteria were first incubated with SP-A in solution and then plated on Müller-Hinton agar supplemented with different antibiotics, the synergy between SP-A and PMB was lost (data not shown). Given the poor diffusion of SP-A in agar, these data suggest that the observed synergy is based on the SP-A/PMB interaction in solution.

**Figure 4:**
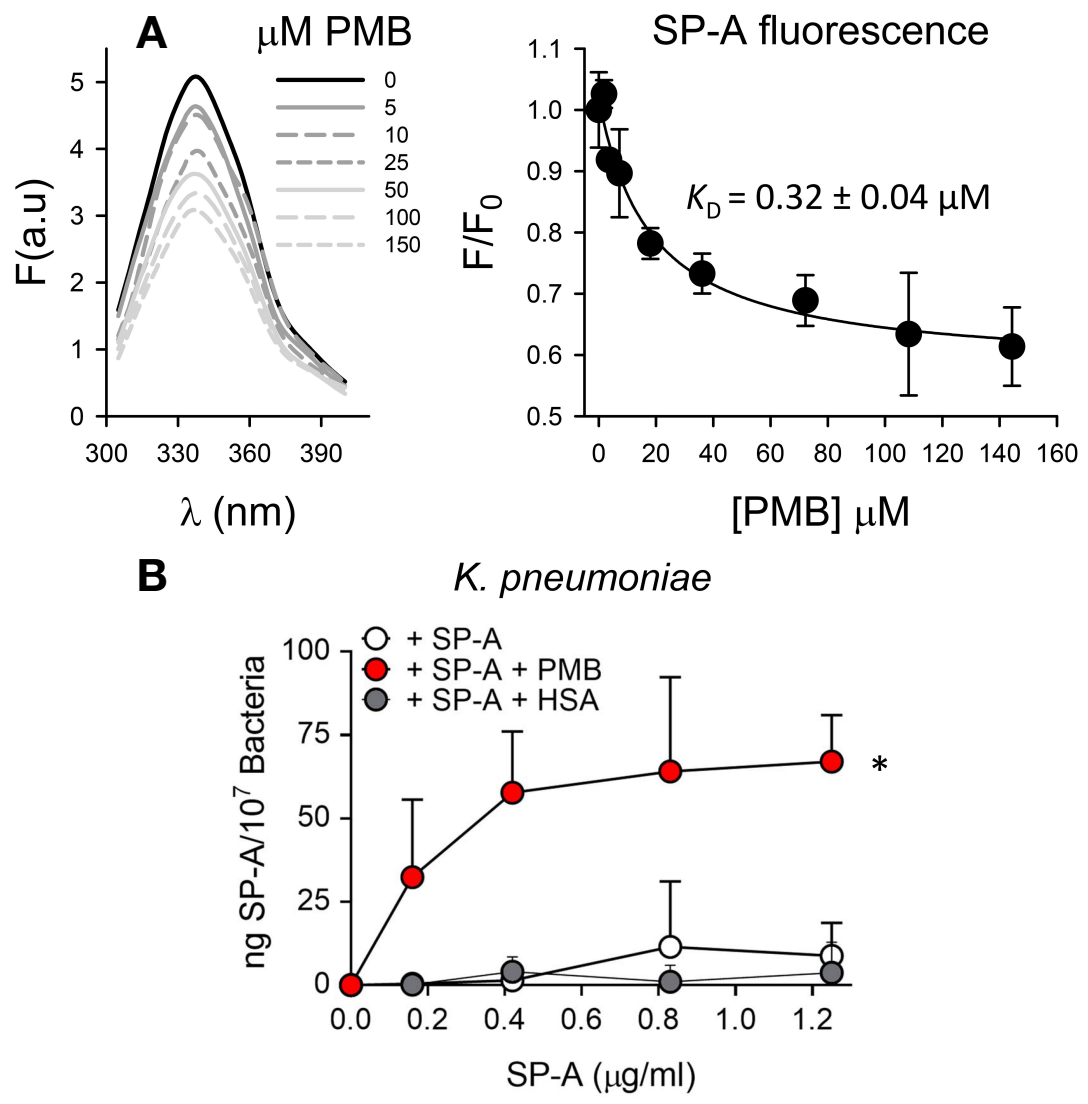
SP-A and polymyxin B interact in a dose-dependent manner, allowing binding of SP-A to *K pneumoniae*. **(A)** (Left) Tryptophan fluorescence emission spectra of SP-A (15 nM) (10 μg/ml) were measured with or without increasing concentrations of PMB (0-150 μM; 0-180 μg/ml) at 25°C in 5 mM Tris-HCl buffer (pH 7.4) containing 150 mM NaCl. SP-A samples (with and without PMB) and blank samples (PMB alone) were excited at 295 nm and the emission spectra recorded from 300 to 400 nm. One representative experiment of four is shown. (Right) Results are expressed as F/F_0_, where F and F_0_ are the corrected emission intensities at 337 nm in the presence and absence of PMB. Results are means ± SD of four experiments. **(B)** Binding capacity of biotinylated SP-A to *K pneumoniae* (10^7^ CFU) in the presence and absence of PMB (5 μg/ml) or HSA (5 μg/ml). The concentration of biotinylated SP-A associated with *K pneumoniae* was measured by a solid phase binding assay. Data are means ± SD of three independent experiments with three biological replicates. A value of *p < 0.05 was obtained for the one-way ANOVA.

We first determined the potential interaction of SP-A and PMB in solution by monitoring changes in the intrinsic fluorescence of SP-A after PMB binding (**Figure 4A**). The fluorescence of SP-A is dominated by the contribution of its two conserved tryptophan residues at the COOH-terminal end of the protein (50). Addition of increasing concentrations of PMB (0 to 150 μM) resulted in a significant PMB concentration-dependent decrease in the amplitude of the fluorescence emission spectrum of SP-A, without any shift in the wavelength of the emission maxima (**Figure 4A**, left). The estimated dissociation constant (*K*_D_) for SP-A/PMB interaction was 0.32 ± 0.04 µM (**Figure 4A**, right), and the Hill coefficient value was greater than 1, indicating a positive cooperative binding.

We next evaluated whether SP-A interaction with PMB allows SP-A binding to *K. pneumoniae* by solid-phase binding assay (**Figure 4B**). SP-A alone did not bind to *K. pneumoniae*, not even in the presence of HSA. However, SP-A was able to bind this respiratory pathogen in the presence of PMB in a dose-dependent manner. Therefore, these results indicate that, through a protein-lipopeptide interaction mechanism, PMB facilitates the binding of SP-A to bacteria.

**Figure 5:**
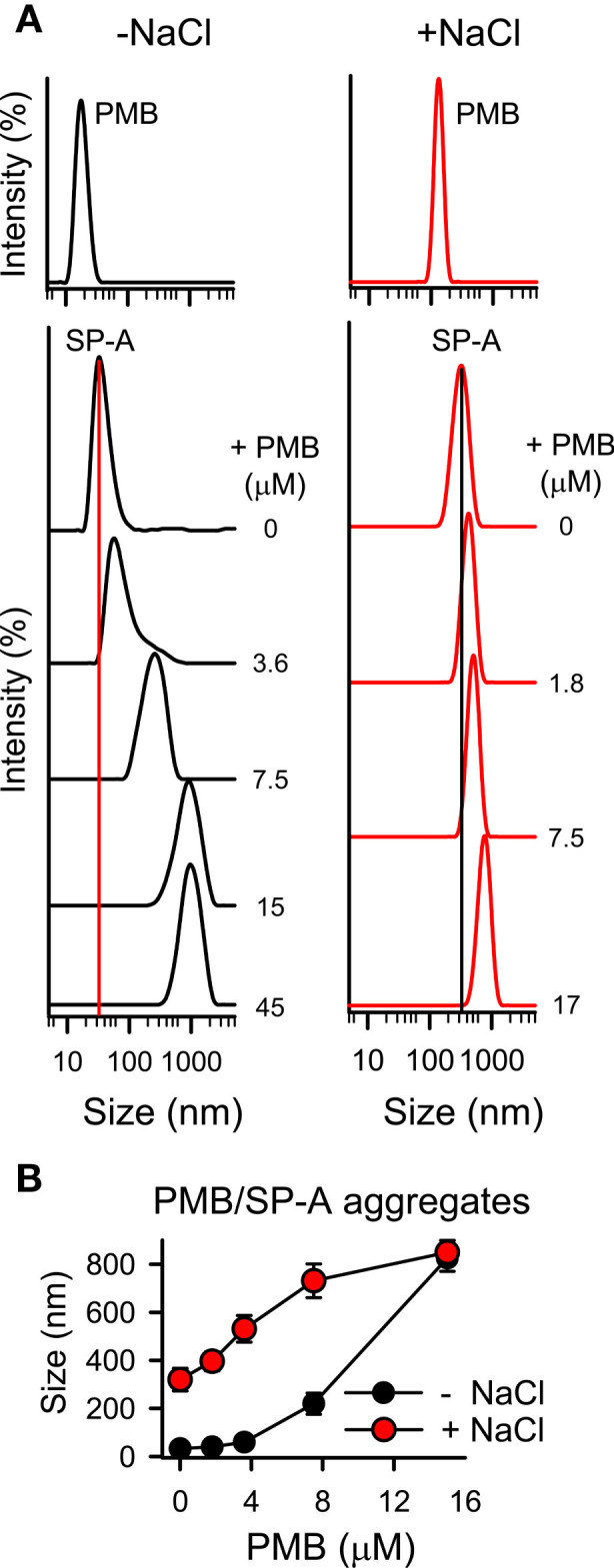
SP-A forms molecular aggregates with polymyxin B. The formation of SP-A and PMB complexes was examined by DLS. **(A)** DLS analysis of the hydrodynamic diameter of SP-A/PMB complexes in 5 mM Tris-HCl buffer (pH 7.4), in the presence or absence of 150 mM NaCl. The y-axis represents the relative intensity of the scattered light; the x-axis represents the hydrodynamic diameter of the particles present in the solution. DLS analyses of SP-A (15 nM) and PMB (15 μM) particles alone in both buffers are shown in the upper graphs. One representative experiment of four is shown. **(B)** Formation of SP-A/PMB aggregates is independent of salts. The results are the mean ± SD of four independent experiments, each in triplicate.

To determine if there are changes in the size of the particles after the interaction of SP-A with PMB in solution, we performed dynamic light scattering in the absence and presence of salts to further determine the role of NaCl in the interaction between positively charged PMB particles and SP-A, a protein rich in negatively charged amino acids, whose isoelectric point varies between pH 4.5 and 5.2 (63). **Figure 5A** shows that addition of PMB to SP-A at neutral pH caused a PMB concentration-dependent increase of SP-A hydrodynamic size, both in the presence and absence of salts, resulting in the formation of SP-A-PMB aggregates of 800-900 nm (**Figure 5B**). **Figure 5A** also shows that PMB alone exhibited a unique peak (independent of concentration) corresponding to particles with a hydrodynamic diameter of 20 ± 3, in the absence of salts, and 150 ± 30 nm, in the presence of 150 mM NaCl (**Figure 5**, upper graphs). On the other hand, SP-A alone showed a hydrodynamic diameter of 34 ± 5 nm in the absence of salts, as previously described (51). In the presence of 150 mM NaCl, SP-A self-aggregates, as previously described (64) with a major peak at 320 ± 30 nm (30) (**Figure 5A**).

### Synergistic antimicrobial activity of SP-A with polymyxin B and colistin

**Figure 6:**
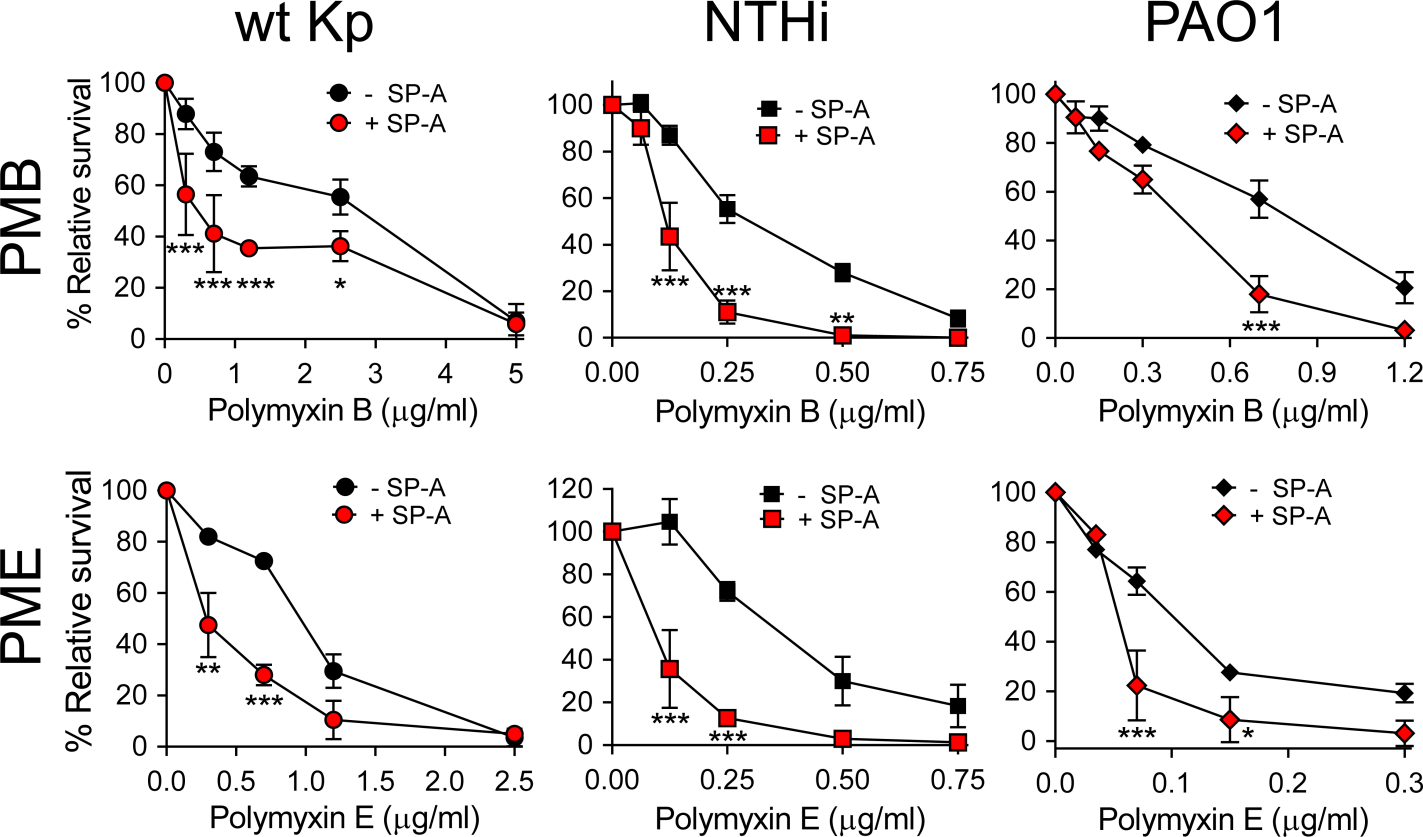
SP-A acts synergistically with polymyxin B and colistin (PME) against Gram-negative bacteria. 10^5^ CFUs/ml of bacteria (*K. pneumoniae*, NTHi, and *P. aeruginosa* O1) were incubated with different concentrations of PMB (upper graphs) and PME (lower graphs) in the absence or presence of SP-A (100 μg/ml) in 10 mM phosphate, 1% TSB, and 100 mM NaCl buffer (pH 7.4) for 1 h at 37°C. Bacteria were then plated on LB agar (*Kp* and PAO1) or sBHI agar (NTHi) for CFU counting. Results are shown as a percentage of relative survival compared to untreated bacteria. Data are means ± SD of four independent experiments, with three biological replicates. Results were statistically analyzed by one-way ANOVA followed by the Bonferroni multiple-comparison test. *p < 0.05, **p < 0.01, and ***p < 0.001 when comparing SP-A-treated with SP-A-untreated *K. pneumoniae* strains.

We next assessed whether SP-A/polymyxin complexes increase the antimicrobial activity of this lipopeptide antibiotic against *K. pneumoniae* K2, non-typable *H. influenzae* (NTHi), and *P. aeruginosa*, which are resistant to SP-A. For these experiments we use polymyxin B and E (colistin), both of which are available for clinical use. Colistin differs from polymyxin B in the amino acid at R6 position of the cyclic heptapeptide part (i.e., residues R4–R10), which is D-phenylalanine in PMB and D-leucine in PME (35). **Figure 6** shows that when bacteria were incubated with SP-A (at concentrations within the ranges found in the alveolar fluid of human lungs) (65) and increasing concentrations of PMB (**Figure 6**, upper panels) and colistin (PME) (**Figure 6**, lower panels), SP-A significantly increased polymyxin bactericidal activity against the three pathogens. Therefore, we demonstrated that human SP-A/polymyxin complexes exhibit significantly increased antimicrobial activity relative to PMB and PME alone.

### Interaction and synergy of SP-A with nontoxic polymyxin nonapeptide

Given that SP-A is a lipid binding protein (63), we determined the role of the lipid moiety of this lipopeptide antibiotic in the interaction with SP-A using polymyxin B nonapeptide (PMBN), a PMB derivative that lacks the fatty acid tail. PMBN maintains polymyxin B’s ability to bind to Gram-negative bacteria and disturb to some extent the outer membrane of these bacteria. However, PMBN alone does not have bactericidal activity (38, 39).

**Figure 7:**
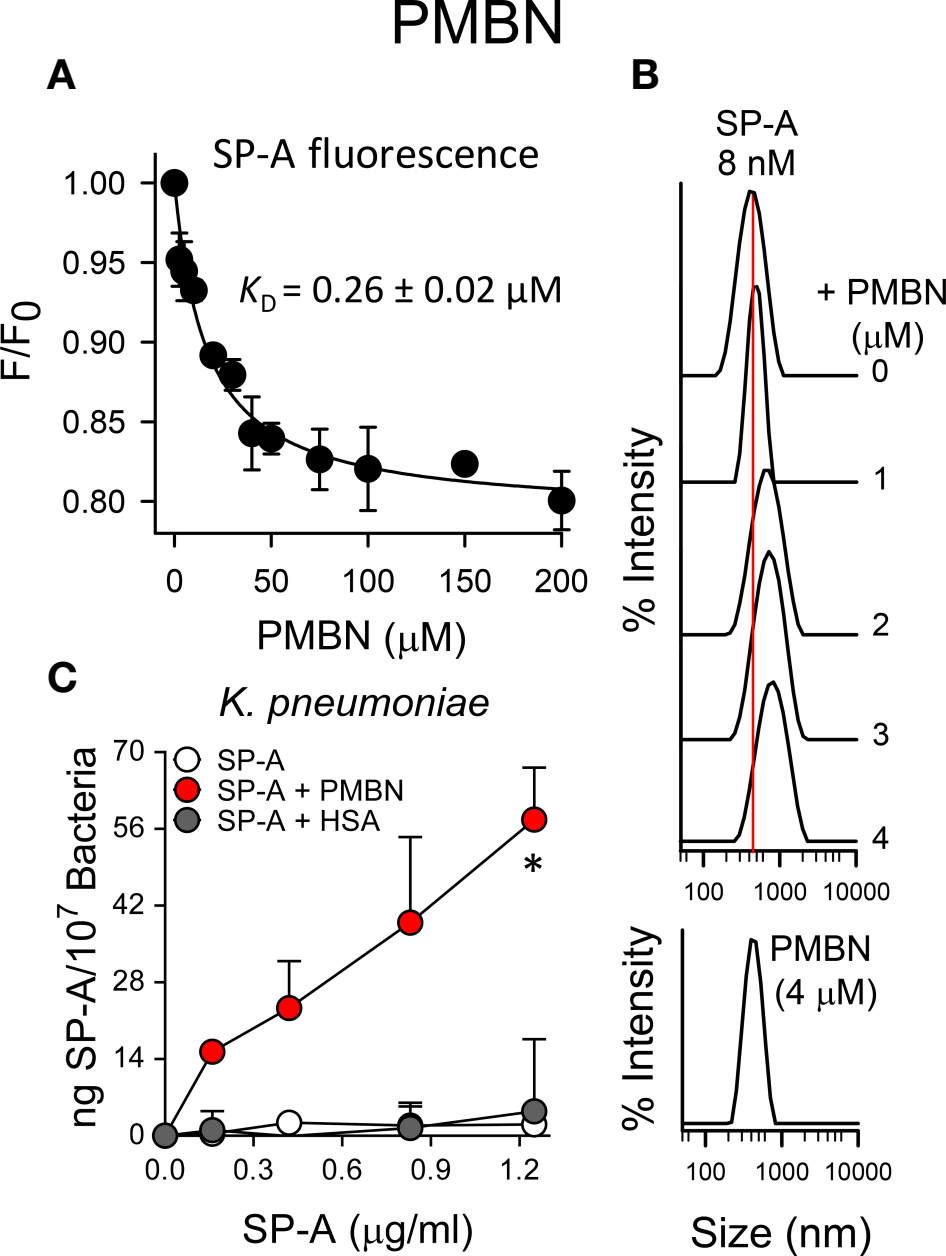
SP-A and PMBN interact in a dose-dependent manner, forming molecular aggregates that allow binding of SP-A to *K pneumoniae*. **(A)** Binding of SP-A and PMBN was examined by changes in intrinsic fluorescence emission spectra of SP-A (15 nM; 10 μg/ml)) by addition of increasing concentrations of PMBN (0 to 200 µM; 0-192 μg/ml)) at 25°C in 5 mM Tris-HCl buffer (pH 7.4) containing 150 mM NaCl. Results are expressed as F/F_0_, where F and F_0_ are the corrected emission intensities at 337 nm in the presence and absence of PMBN. Results are means ± SD of four experiments. **(B)** DLS analysis of the hydrodynamic diameter of SP-A/PMBN complexes in 5 mM Tris-HCl, 150 mM NaCl buffer (pH 7.4). The y-axis represents the relative intensity of the scattered light; the x-axis denotes the hydrodynamic diameter of the particles present in the solution. DLS analyses of SP-A and PMBN particles alone are also shown. One representative experiment of four is shown. **(C)** Binding capacity of biotinylated SP-A to *K pneumoniae* (10^7^ CFU) in the presence and absence of PMBN (5 μg/ml) or HSA (5 μg/ml). The concentration of biotinylated SP-A associated with *K pneumoniae* was measured by a solid phase binding assay and expressed as total nanograms of SP-A/10^7^ bacteria. The results are the mean ± SD of three independent experiments with three biological replicates. A value of *p < 0.05 was obtained for the one-way ANOVA.

**Figure 7A** shows that SP-A interacted with PMBN with an estimated dissociation constant (*K*_D_ = 0.26 ± 0.04 µM) similar to that obtained for the lipopeptide antibiotic. The binding of SP-A to PMBN also resulted in the formation of molecular aggregates similar in hydrodynamic size to those formed with PMB, as determined by dynamic light scattering (**Figure 7B**). In addition, the formation of SP-A/PMBN aggregates, through a protein-protein interaction mechanism, facilitates the binding of SP-A to *K. pneumoniae* to which SP-A alone does not bind (**Figure 7C**).

**Figure 8:**
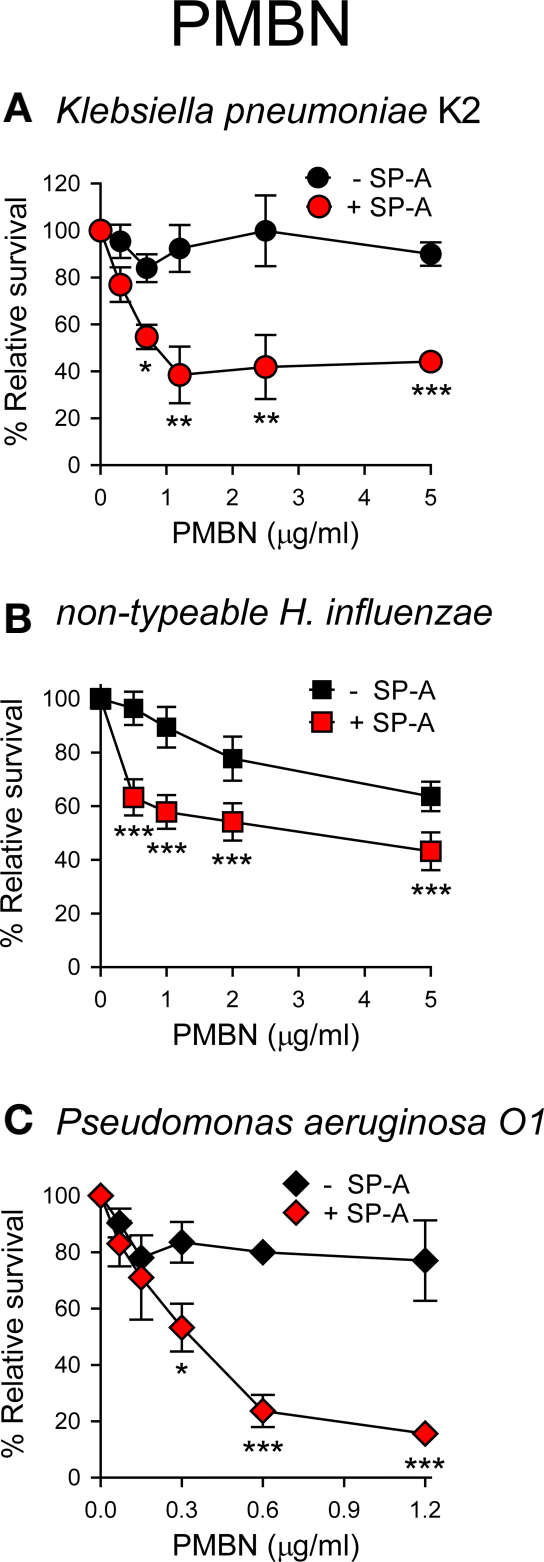
SP-A/PMBN complexes kill Gram-negative bacteria. 10^5^ CFUs/ml of *K pneumoniae* **(A)**, NTHi **(B)**, and *P. aeruginosa* O1 **(C)** were incubated with increasing concentrations of PMBN in the absence or presence of SP-A (100 μg/ml for *Kp* and PAO1; 25 μg/ml for NTHi) for 1 h at 37°C. Bacteria were then plated on LB agar (*Kp* and PAO1) or sBHI agar (NTHi) for CFU counting. Results are shown as a percentage of relative survival compared to untreated bacteria. Data are means ± SD of four independent experiments, with three biological replicates. Results were statistically analyzed by one-way ANOVA followed by the Bonferroni multiple-comparison test. *p < 0.05, **p < 0.01, and ***p < 0.001 when bacteria treated with SP-A+PMBN were compared against bacteria treated with PMBN.

Next, we sought to ascertain whether the formation of SP-A/PMBN aggregates confers new antimicrobial properties, including the ability to kill pathogenic Gram-negative bacteria that are otherwise resistant to either SP-A or PMBN alone. We found that SP-A/PMBN complexes were able to kill *K. pneumoniae*, non-typable *H. influenzae*, and *P. aeruginosa* (**Figure 8**), indicating that the combination of nontoxic PMBN and SP-A could be used as a new antimicrobial against respiratory Gram-negative infections.

### Bacterial membrane permeabilization

**Figure 9:**
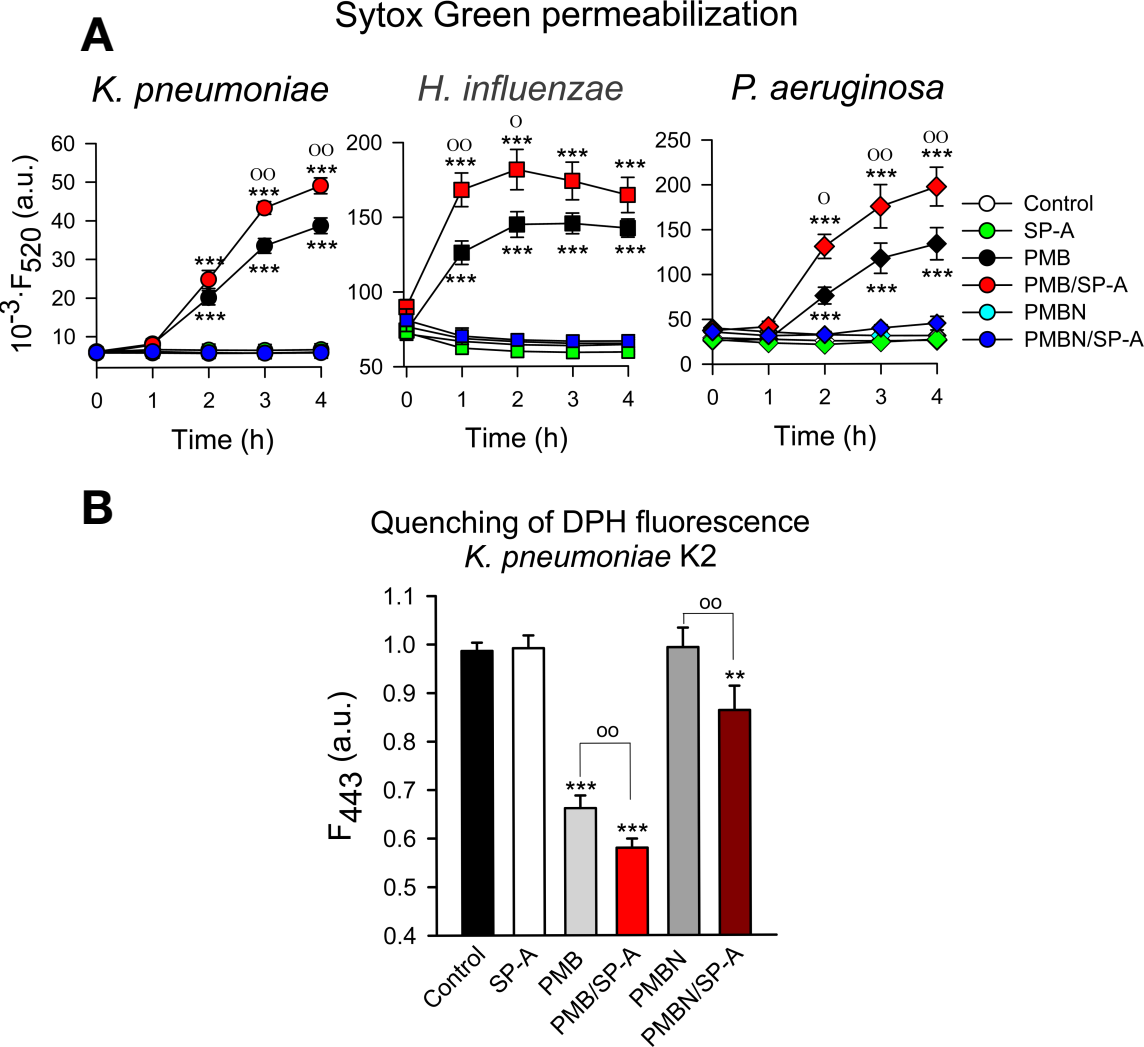
Membrane integrity of Gram-negative bacteria. **(A)** 10^7^ CFU/ml of **(*K*)** *pneumoniae*, NTHi, and PAO1 were incubated with polymyxins (PMB or PMBN) and/or SP-A (100 μg/ml) in the presence of Sytox Green, and the change in the fluorescence of the dye was recorded as a function of time. The experiments were conducted at 37°C. PMB concentrations: 1 μg/ml for *K pneumoniae*; 0.5 μg/ml for NTHi and PAO1. PMBN concentrations: 1 μg/ml for *K pneumoniae*; 2 μg/ml for NTHi; 0.5 μg/ml for PAO1. **(B)** 10^7^ CFU/ml of *K pneumoniae* were incubated with or without PMB (1μg/ml), PMBN (1μg/ml) and/or SP-A (100 μg/ml) in the presence of 1,6-diphenyl-1,3,5-hexatriene (DPH), and the change in the fluorescence of DPH was recorded as a function of time. Data shown correspond to the fluorescence of DPH after treatment with SP-A and/or polymyxins for 30 min. Experiments were performed at 37°C. The results are the mean ± SD of three independent experiments, each in triplicate. Results were statistically analyzed by one-way ANOVA followed by the Bonferroni multiple-comparison test: **p < 0.01; ***p < 0.001 compared to untreated bacteria (control); ^о^ *p <* 0.05; ^оо^ *p* < 0.01 when bacteria treated with SP-A+polymyxin (PMB or PMBN) were compared with bacteria treated with PMB or PMBN alone.

The ability of SP-A, PMB, PMBN, and combinations thereof to permeabilize bacterial membranes of *K. pneumoniae*, NTHi and *P. aeruginosa* was assessed by using the fluorescent dye Sytox Green, whose fluorescence is enhanced upon binding to DNA once the bacterial cytoplasmic membrane is compromised (66). Addition of SP-A alone did not statistically affect the fluorescence of the dye (**Figure 9A**). These results align with the absence of SP-A binding to any of these pathogens and its incapacity to kill these bacteria on its own. However, PMB significantly increased the dye’s fluorescence, consistent with the bacterial membrane permeabilization properties of PMB (36, 37). Moreover, bacteria treated with SP-A+PMB showed significantly greater Sytox Green permeabilization than those treated with PMB alone. Thus, the formation of SP-A/PMB aggregates significantly increases PMB-induced bacterial membrane permeabilization and consequent bacterial death.

In contrast to SP-A/PMB aggregates, SP-A/PMBN aggregates did not promote permeabilization of Sytox Green through Gram-negative bacterial membranes, nor did PMBN alone (**Figure 9A**). This suggests that the synergistic microbicidal activity of SP-A/PMBN aggregates could be related to mechanisms of action other than permeabilization of the cytoplasmic membrane. To determine if SP-A, PMB, PMBN, and combinations thereof alter the integrity of *K. pneumoniae* membranes, we measured the fluorescence of 1,6-diphenyl-1,3,5-hexatriene (DPH), a fluorescent dye that incorporates into the hydrophobic core of lipid bilayers. DPH has a low quantum yield and a very short lifetime when exposed to water. Thus, its fluorescence is sensitive to the amount of water that penetrates the lipid bilayer (67). **Figure 9B** shows that SP-A or PMBN alone did not affect the fluorescence of the probe, while PMB alone and PMB/SP-A and PMBN/SPA mixtures significantly decreased the dye’s fluorescence, indicating the presence of water molecules in the hydrophobic microenvironment of *K. pneumoniae* membranes. The effect of PMB/SP-A complexes on membrane alteration was significantly greater than that of PMB. Likewise, the effect of PMBN/SP-A on the integrity of *K. pneumoniae* membranes was significantly greater than that of PMBN, which did not exhibit any effect.

### Bacterial lipopolysaccharide aggregation.

**Figure 10:**
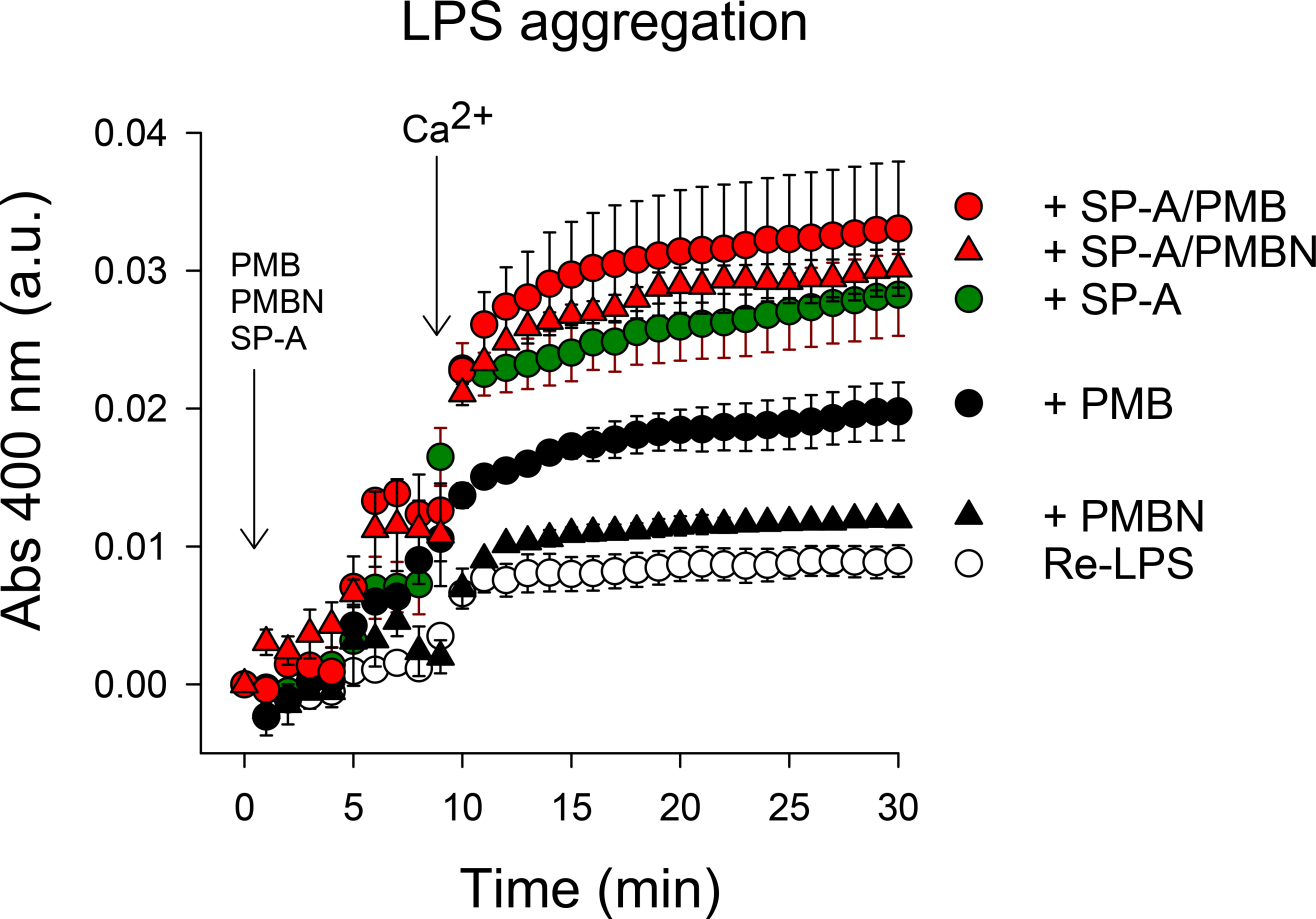
LPS aggregation induced by SP-A, PMB, PMBN, and combinations thereof. Ca^2+^-dependent Re-LPS aggregation was studied by measuring the change in absorbance at 400 nm in a Beckman DU-800 spectrophotometer. Re-LPS was prepared at a final concentration of 40 μg/ml in 5 mM Tris-HCl buffer, pH 7.4, containing 150 mM NaCl and 0.1 mM EDTA. The final concentration of SP-A and polymyxins was 20 μg/ml (30 nM) SP-A, 1.5 μg/ml (1.08 µM) PMB, and 2.5 μg/ml (2.59 µM) PMBN. The concentration of Ca^2+^ was 2.5 mM. Results are the mean ± SD of three experiments.

Polymyxins and SP-A are LPS binding peptides and induce LPS aggregation (16, 33, 34, 57, 68). Thus, we analyzed Re-LPS aggregation induced by SP-A, PMB, PMBN, and combinations thereof (**Figure 10**). We found that PMB/SP-A and PMBN/SP-A complexes exhibited higher LPS aggregation activity than PMB or PMBN alone at the concentrations analyzed.

Several lines of evidence have shown that the carbohydrate recognition domain (CRD) of SP-A is involved in its binding to the lipid A moiety of LPS and to dipalmitoylphosphatidylcholine (DPPC), a main pulmonary surfactant component (69–71). Thus, these experiments suggest that PMB (or PMBN) binds to SP-A at sites other than the lipid-binding site on the globular heads of SP-A. To confirm this assumption, we determined the ability of SP-A to bind and aggregate DPPC vesicles in the presence and absence of PMB, which does not bind zwitterionic phosphatidylcholine (72). **Supplementary Figure 2** shows that PMB alone, in excessive concentration, did not aggregate DPPC vesicles, but SP-A/PMB complexes induced DPPC aggregation almost like SP-A alone. Together, these data indicate that the binding of PMB to SP-A does not interfere with the ability of SP-A to bind and aggregate lipids and that PMB/SP-A interaction might not occur *via* the globular domains of SP-A.

### Direct antimicrobial activity of rfhSP-A against Gram-negative bacteria

**Figure 11:**
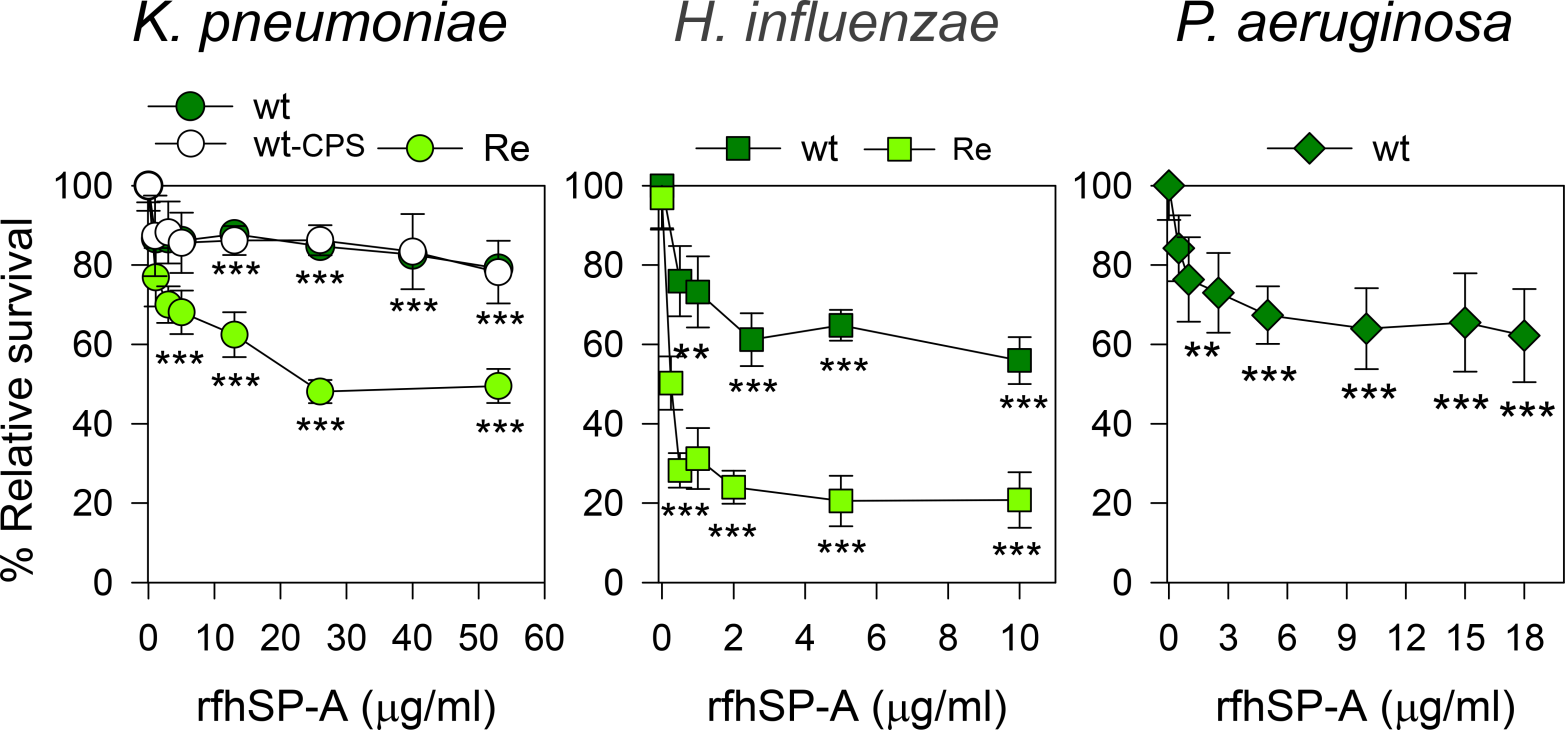
rfhSP-A kills Gram-negative bacteria. 10^5^ CFUs/ml of different strains of Kp, NTHi, or PAO1 were incubated with increasing concentrations of rfhSP-A in 10 mM phosphate buffer (pH 7.4) containing 1% TSB and 100 mM NaCl for 1 h at 37°C. Bacteria were then plated on LB agar (Kp and PAO1) or sBHI agar (NTHi) for CFU counting. Results are shown as a percentage of relative survival compared to untreated bacteria. Importantly, SP-A had no effect on the survival of these pathogens at molar concentrations equivalent to or greater than the molar concentrations of rfhSP-A. Data are means ± SD of three independent experiments, with three biological replicates. Experiments were performed with both polymyxin-agarose treated and untreated rfhSP-A, to remove LPS contamination, with identical results. Results were statistically analyzed by one-way ANOVA followed by the Bonferroni multiple-comparison test. **p < 0.01, and ***p < 0.001 when bacterial strains treated with rfhSP-A were compared with those not treated with rfhSP-A.

We next evaluated the bactericidal activity of a recombinant trimeric fragment of human SP-A1 (rfhSP-A), which lacks the N-terminal domain and the collagen domain (**Figure 11**). In contrast to native supratrimeric SP-A, a small trimeric fragment of the protein (containing three globular heads) was able to kill also wt strains of respiratory pathogens (*K. pneumoniae*, NTHi, and *P. aeruginosa*). We found that rfhSP-A possesses identical antimicrobial activity against encapsulated and non-encapsulated wt strains of *K. pneumoniae*, indicating that CPS is not relevant if *K. pneumoniae* contains whole LPS glycoconjugates. However, the bactericidal activity of rfhSP-A was greater with the non-capsulated *K. pneumoniae* mutant expressing deep-rough lipopolysaccharide (Re-Kp-CPS) than with the non-capsulated wild-type strain (**Figure 11**). On the other hand, the rfhSP-A-induced killing of the Re-NTHi mutant strain expressing only KDO residues at its oligosaccharide moiety of LOS was much greater than that of the wild-type NTHi strain expressing long chains of LOS glycoconjugates. These results suggest that the umbelliform-shaped structure of native SP-A impedes SP-A binding to respiratory Gram-negative pathogens. Conversely, smaller fragments of SP-A, which retain the carbohydrate recognition domain and lipid binding site in their globular heads, bind to these pathogens and show bactericidal properties.

Although the small rfhSP-A protein binds and partially kills *P. aeruginosa*, NTHi, and *K. pneumoniae*, rfhSP-A is unable to aggregate not only wt PAO1 and *K. pneumoniae*, but also non-capsulated deep rough *K. pneumoniae* strains, which are aggregated by SP-A (**Supplementary Figure S2**). The inability of rfhSP-A to induce bacterial aggregation is consistent with the fact that SP-A requires an oligomeric assembly to induce aggregation of its different ligands (34).

### Loss of interaction and synergy between rfhSP-A and polymyxin B

**Figure 12:**
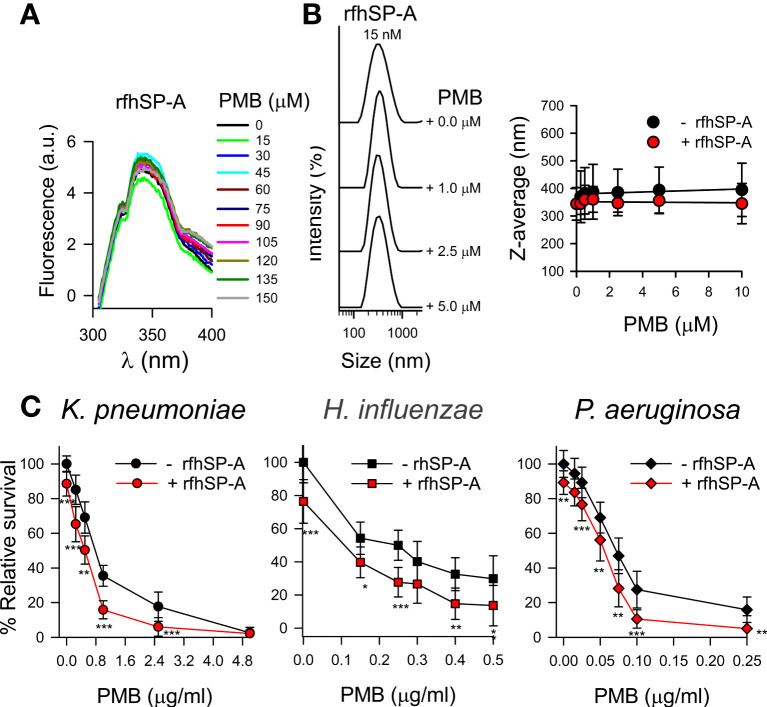
rfhSP-A does not interact with PMB and in combination with PMB increases the killing of Gram-negative bacteria. **(A)** Tryptophan fluorescence emission spectra of rfhSP-A (13 μg/ml) were measured with or without increasing concentrations of PMB (0-150 μM; 0-180 μg/ml) at 25°C in 5 mM Tris-HCl buffer (pH 7.4) containing 150 mM NaCl. Samples were excited at 295 nm and the emission spectra recorded from 300 to 400 nm. One representative experiment of four is shown. **(B)** (Left) Addition of increasing concentrations of PMB (ranging from 0 to 5 μM) to a solution containing a constant concentration of rfhSP‐A (17.5 nM) in 5 mM Tris-HCl, 150 mM NaCl, buffer (pH 7.4) did not change the particle size of either SP‐A or PMB. One representative experiment of four is shown. (Right) Dependence of Z-average on the PMB concentration in the presence or absence of rfhSP-A (17.5 nM). **(C)** 10^5^ CFUs/ml of bacteria (*K. pneumoniae*, NTHi, and *P. aeruginosa* O1) were incubated with different concentrations of PMB in the absence or presence of rfhSP-A (1 μg/ml) (17.5 nM) in 10 mM phosphate, 1% TSB, and 100 mM NaCl buffer (pH 7.4) for 1 h at 37°C. Bacteria were then plated on LB agar (*Kp* and PAO1) or sBHI agar (NTHi) for CFU counting. Results are shown as a percentage of relative survival compared to untreated bacteria. The results in **(A)** and **(B)** correspond to rfhSP-A treated with polymyxin agarose in 5 mM Tris, 150 mM NaCl, pH 7.4 buffer to remove LPS contamination. In **(C)**, experiments were performed with both polymyxin-agarose treated and untreated rfhSP-A, with identical results. Data are means ± SD of three independent experiments, each in triplicate. Results were statistically analyzed by Student’s t-test. *p < 0.05, **p< 0.01, and ***p < 0.001 when bacteria treated with rfhSP-A+PMB were compared with bacteria treated with PMB alone.

The trimeric recombinant fragment of human SP-A1 did not interact with PMB in solution at physiological concentrations of salts, as determined by intrinsic fluorescence of rfhSP-A after PMB binding (**Figure 12A**) and dynamic light scattering (**Figure 12B**). This suggests that the collagen-like domain and/or the supratrimeric structure of SP-A is essential for SP-A/PMB interaction and the formation of protein-lipopeptide aggregates. In the absence of salts, rfhSP-A weakly interacts with PMB with a *K_D_* of 1.37 ± 0.5 μM.

**Figure 13:**
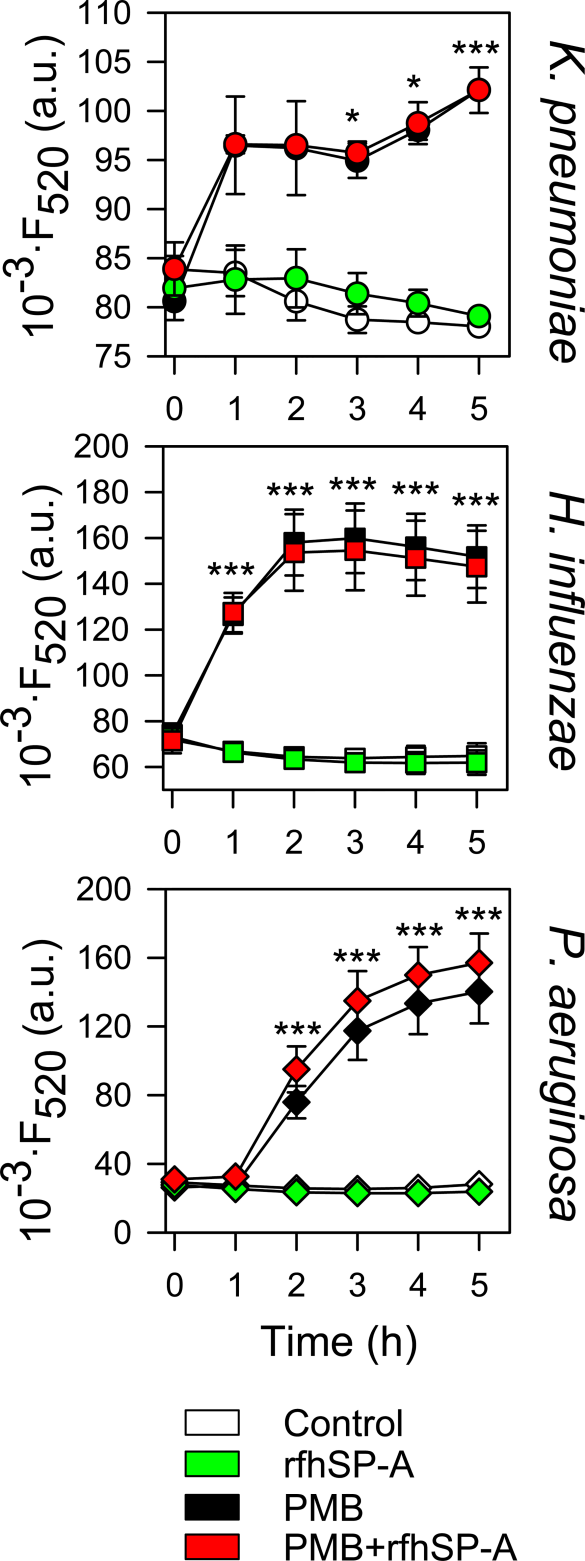
rfhSP-A does not affect the ability of PMB to permeabilize bacterial membranes. 10^7^ CFU/ml of *K. pneumoniae*, NTHi, and PAO1 were incubated with PMB (1 μg/ml for *K. pneumoniae*; 0.5 μg/ml for NTHi and PAO1) and/or rfhSP-A (5 μg/ml) in the presence of Sytox Green, and the change in the fluorescence of the dye was recorded as a function of time. The results are the mean ± SD of three independent experiments, each in triplicate. A *p*-value < 0.001 was obtained for the one-way ANOVA followed by the Bonferroni multiple-comparison test: *p < 0.05 and ***p < 0.001 compared to untreated bacteria (control).

Due to the loss of interaction under physiological conditions, rfhSP-A and PMB did not show synergistic action. However, rfhSP-A added in combination with PMB significantly increased antimicrobial efficacy against *K. pneumoniae*, non-typable *H. influenzae*, and *P. aeruginosa* (**Figure 12C**). The combination of rfhSP-A with other antibiotics against Gram-negative bacteria also significantly improved bactericidal efficacy against *K. pneumonia*e, indicating an additive effect of rfhSP-A with other antibiotics in addition to PMB (**Supplementary Figure 3**). Consistent with the lack of interaction and synergy between rfhSP-A and PMB, the recombinant fragment did not affect the ability of PMB to permeabilize bacterial membranes of Gram-negative bacteria (**Figure 13**). On the other hand, rfhSP-A did not affect bacterial membrane permeabilization on its own, suggesting a different mechanism for its bactericidal activity.

## Discussion

SP-A is secreted into the airway mucosa by type II alveolar epithelial cells and non-ciliated bronchiolar cells, but it is also detected in the trachea, nasal mucosa, and other extrapulmonary mucosal surfaces, where it provides immune protection (17–19). It enhances phagocytosis of pathogens by opsonizing and/or up-regulating expression of the mannose receptor involved in microbial recognition (17, 19, 73, 74). In addition, SP-A can agglutinate some bacteria and has direct microbicidal activity on some microorganisms (20–24). However, reports of direct microbicidal activity of SP-A against clinically relevant Gram-negative bacteria are scarce, and several studies have shown that highly virulent respiratory pathogens are resistant to SP-A (27–31, 75).

In this study, we examined factors involved in the resistance of clinical isolates of *K. pneumoniae* and nontypeable *H. influenzae* to SP-A. Using *K. pneumoniae* 52145 and isogenic mutants with or without capsule and expressing a range of different LPS phenotypes, we show that both the K2 polysaccharide capsule and the underneath LPS glycoconjugates are not recognized by SP-A. SP-A exhibited direct antimicrobial activity (bacterial binding, aggregation, and killing) only against non-capsulated deep rough *K. pneumoniae* strains expressing Rc- and Re-LPS phenotypes. The small number of sugar residues in the rough strains makes it easier for SP-A to recognize the lipid A region of LPS (76, 77). Some lines of evidence suggest that *K. pneumoniae* may undergo phase variation between capsulated and non-capsulated phenotypes since non-capsulated phase variants of *K. pneumoniae* attach to and invade epithelial cells more efficiently (75, 78). SP-A would not have direct microbicidal activity against non-capsulated *Klebsiella* because of its long-chain lipopolysaccharides shield *K. pneumoniae* from SP-A. Interestingly, the macrophage mannose receptor together with SP-A provides protection against *K. pneumoniae* K21a serotype that expresses the Man-α2-Man sequence in its CPS, which is recognized by SP-A and the mannose receptor (27, 75). There is a significant correlation between the presence of this capsular glycoepitope, recognized by SP-A and other C-type lectins, and the low frequency of *K. pneumoniae* K21a in clinical isolates of patients (75).

Nontypeable *H. influenzae* is a non-capsulated Gram-negative bacterium that causes exacerbations in patients with chronic obstructive pulmonary disease (79, 80) and colonizes the lower respiratory tract of patients with neutrophilic asthma (81). NTHi express a lipooligosaccharide (LOS), which lacks the O-specific polysaccharide chains. LOS glycoconjugates are analogous to LPS found in other Gram-negative families, but tend to have short, nonrepeating oligosaccharides. They also share similar lipid A structures. Using clinical isolates of NTHi375 and isogenic mutants with truncated LOS variants, we showed that SP-A exhibited direct microbicidal activity against only the deep rough mutant Re-NTHi strain, which retains only the KDO component of LOS. These results indicate that LOS glycoconjugates of NTHi confer protection against the bactericidal and bacterial aggregation-inducing activities of SP-A.

Our results are consistent with other reports indicating that one of the factors conferring resistance to SP-A in *Bordetella pertussis* is the branched structure of the LPS core (29). For *P. aeruginosa*, several studies have indicated that there are multiple virulence factors that explain its resistance to SP-A: i) reduced expression of the outer membrane protein OprH (a ligand for SP-A) (82); ii) increased expression of glycoconjugates of long-chain LPS (82); and iii) expression of type IV pilus on the cell wall (83). In addition, *P. aeruginosa* secretes elastase and protease IV that degrade SP-A (84, 85). These adaptations of *P. aeruginosa* facilitate bacterial escape from SP-A-mediated phagocytic killing (82).

In contrast to SP-A, rfhSP-A, a trimeric recombinant fragment of the protein, surprisingly showed significant direct microbicidal activity against *K. pneumoniae*, NTHi, and *P. aeruginosa*. The trimeric recombinant fragment lacks the N-terminal domain and most of the collagen domain, which are domains involved in SP-A oligomerization. However, rfhSP-A retains ligand binding sites in the globular domains and the α-helical neck domain. The different functionality of SP-A and rfhSP-A could be related to the smaller size of rfhSP-A, which facilitates the transit of rfhSP-A through the glycoconjugate structures of CPS and LPS, or LOS in the case of NTHi. However, the bactericidal activity of rfhSP-A was higher with the non-encapsulated *K. pneumoniae* mutant expressing Re-LPS (Re-kp-CPS) and with the Re-NTHi mutant than with non-encapsulated *K. pneumoniae* and NTHi, which express long-chain glycoconjugate structures. These data indicate that these glycoconjugate assemblies also confer protection against the bactericidal action of rfhSP-A.

Direct *in vitro* killing of *K. pneumoniae*, NTHi, and *P. aeruginosa* by native human SP-A is negligible (27–31). It is possible that defense AMPs in the alveolar fluid act synergistically or cooperatively with SP-A in eliminating these pathogens, as we recently demonstrated for synergic SP-A/SP-B^N^ interaction (30, 31). It has recently been shown that the combined use of AMPs and antibiotics can kill drug‐resistant pathogens, reduce antibiotic resistance, and significantly improve the therapeutic effects of antibiotics (86). In this study, we examined the potential cooperation of SP-A and its trimeric recombinant fragment with several antibiotics, including polymyxin B, azithromycin, tetracycline, and ciprofloxacin against Gram-negative bacteria. We chose these antibiotics because they have different mechanisms of action and different targets on Gram-negative bacteria (58). We found that SP-A only acted cooperatively with polymyxin B. SP-A bound PMB in solution with an estimated *K*_D_ of 0.32 ± 0.04 µM. Binding occurs through SP-A recognition of the polycationic peptide ring of polymyxin, since SP-A bound PMBN, a derivative of PMB lacking the fatty acid tail (33), with the same affinity. The binding of SP-A to PMB or PMBN resulted in the formation of macromolecular aggregates in the presence of physiological ionic strength. The formation of SP-A/PMB and SP-A/PMBN complexes facilitated the binding of SP-A to *K. pneumoniae*, to which SP-A alone does not bind.

We found that SP-A acted synergistically with PMB and colistin (PME) against *K. pneumoniae*, NTHi, and *P. aeruginosa*. Thus, SP-A potentiates the bactericidal action of two potent but relatively cytotoxic antibiotics. Since toxicity is dose-dependent, the potential use of these synergistic combinations offers a new strategy to improve the clinical utility and safety of polymyxins. Importantly, the interaction between SP-A and PMBN conferred significant bactericidal properties to the SP-A/PMBN complex against *K. pneumoniae*, NTHi, and *P. aeruginosa*, which are resistant to both PMBN and SP-A, individually. PMBN has no bactericidal activity but is still capable of binding to the outer membrane of Gram-negative bacteria, causing membrane disturbances that make Gram-negative bacteria susceptible to various hydrophobic antibiotics (38, 39). The markedly lower toxicity of the PMBN molecule compared to that of PMB suggests that the use of synergistic combinations of SP-A/PMBN could be a promising strategy to treat respiratory infections by multidrug-resistant bacteria.

In Gram-negative infections, killing of bacteria by antibiotics and antimicrobials from the innate immune system is accompanied by the release of LPS, an endotoxin that causes high inflammation. Here we show the ability of SP-A/PMB and SP-A/PMBN complexes to aggregate LPS. Aggregation of LPS particles reduces LPS toxicity (87) and facilitates its phagocytosis by alveolar macrophages (88). Aggregation of LPS by SP-A or PMB blocks LPS interaction with its receptor complex, which reduces proinflammatory cytokine production (16, 57). Furthermore, in the lung, LPS promotes destabilization and alteration of the biophysical activity of pulmonary surfactant (89), and SP-A and PME act as a scavenger of LPS, protecting pulmonary surfactant from the inhibitory effects of LPS (90).

In contrast to SP-A, rfhSP-A did not bind to PMB in solution at physiological salt concentrations, suggesting that the N-terminal domain, the collagen domain, and/or the supratrimeric structure of SP-A are essential for polymyxin binding. Consistent with their lack of interaction, rfhSP-A and PMB did not show synergistic action. However, addition of rfhSP-A in combination with PMB significantly increased antimicrobial efficacy against *K. pneumoniae*, non-typable *H. influenzae*, and *P. aeruginosa*. Moreover, rfhSP-A added in combination with azithromycin, tetracycline, and ciprofloxacin significantly enhanced the killing of *K. pneumonia*e, indicating an additive effect of rfhSP-A with other antibiotics in addition to PMB. Another important difference between SP-A and rfhSP-A is the inability of rfhSP-A to induce bacterial aggregation or LPS aggregation (91), which is consistent with the fact that SP-A requires an oligomeric assembly to induce aggregation of its different ligands (34).

In relation to the possible site of interaction between SP-A and PMB, SP-A binds to the polycationic peptide ring of polymyxins, and the binding of PMB (or PMBN) to SP-A could occur at domains other than the globular domain or the α-helical domain, since rfhSP-A does not bind PMB. The fact that the binding of PMB to SP-A does not interfere with the ability of SP-A to bind and aggregate LPS or DPPC particles by its globular domains strongly suggests that PMB (or PMBN) binds to SP-A at sites other than the lipid-binding site on the globular heads of SP-A.

Regarding the mechanisms of bactericidal action of SP-A/polymyxin complexes, we show that the formation of SP-A/PMB aggregates significantly increased PMB-induced bacterial membrane permeabilization and consequent bacterial death of *K. pneumoniae*, NTHi, and *P. aeruginosa*. PMB-dependent alteration of the outer bacterial membrane could facilitate the binding of SP-A to the lipid A portion of LPS, allowing SP-A-mediated extraction of LPS molecules from the membrane (77). We propose a model in which SP-A/PMB aggregates bind onto the bacterial surface and cover it like a carpet. This high local protein concentration would facilitate the formation of lipoprotein aggregates, resulting in a loss of membrane lipids, leading to membrane leakage and permeabilization. Polymyxin appears to promote contacts between the periplasmic leaflets of the bacterial inner and outer membranes, which would promote lipid exchange between the inner and outer membrane and osmotic imbalance. In contrast to SP-A/PMB aggregates, SP-A/PMBN aggregates did not promote permeabilization of the Sytox Green fluorescent dye through Gram-negative bacterial membranes. However, SP-A/PMBN mixtures, like SP-A/PMB, significantly altered the hydrophobic microenvironment of *K. pneumoniae* membranes, as detected by DPH fluorescence. These data suggest that the interaction of SP-A/PMBN aggregates with the bacterial surface could decrease the dense packing of the bacterial outer membrane and produce transient defects that facilitate the passage of the cyclic peptide through the outer and cytoplasmic membrane. Polymyxins can inhibit crucial intracellular processes such as cell division and bacterial respiration (37). Furthermore, polymyxins bind to bacterial ribosomes to inhibit protein synthesis (37).

Our results suggest that the synergistic antimicrobial activity of SP-A and polymyxin combinations depends on the formation of protein aggregates, since rfhSP-A, which lacks domains necessary for oligomerization and self-aggregation, did not form aggregates with PMB in physiological solutions. Consequently, rfhSP-A did not show synergistic action with PMB. Many antimicrobial proteins and peptides form protein aggregates related to their bactericidal activity, such as human LL-37 (92), protegrine-1 (93), dermaseptin S9 (94), temporins B and L (95), SP-B^N^ at acidic pH (30), and the SP-A/SPB^N^ complex at neutral pH (30). However, the role of protein aggregates in direct killing of bacterial cells remains to be fully determined.

PMB and colistin are available for aerosol administration in humans. Colistin/SP-A complexes administered by nebulization could be beneficial in the treatment of Gram-negative bacterial infections and in LPS neutralization. Moreover, SP-A/colistin administration by nebulization could have the advantage of placing SP-A/polymyxin complexes in a strategic location at the air-liquid interface, which is the first line of defense against inhaled pathogens and endotoxins entering the alveoli. This location could prevent the transport of polymyxin to plasma through the alveolar-capillary barrier, since high concentrations of polymyxin in plasma are related to nephrotoxicity (35, 38, 96). Consistent with this hypothesis, clinical evidence indicates that nebulized colistimethate sodium is effective in treating lower respiratory tract infections caused by multidrug-resistant Gram-negative bacteria without increasing plasma colistin concentration (97). Importantly, it has recently been shown, in an animal model of pneumonia, that instillation of colistin mixed with exogenous pulmonary surfactant increases its bactericidal effect compared to instillation of colistin alone, suggesting that lung surfactant may serve as a vehicle to facilitate the efficient spread of colistin through the airways (98). We suggest that therapeutic SP-A/colistin complexes mixed with exogenous surfactant might be beneficial in treating resistant Gram-negative bacterial infections and protecting pulmonary surfactant from the inhibitory effects of LPS (90).

The combined use of SP-A and colistin to enhance the therapeutic effects of this antibiotic requires the development of recombinant SP-A as a clinical therapeutic agent. The production of full-length oligomeric recombinant SP-A is expensive due to low expression yields in mammalian systems and difficulties in molecular characterization and handling of this protein (33, 34). Producing a small trimeric fragment of SP-A as an alternative to the full-length oligomeric protein has clear advantages in terms of ease of production, handling, and cost (40, 41). In this study, we show that rfhSP-A exhibited significant direct microbicidal activity against *K. pneumoniae*, NTHi, and *P. aeruginosa.*The mechanism of bactericidal action of rfhSP-A against these Gram-negative bacteria is unknown. rfhSP-A did not affect bacterial membrane permeability of these pathogens, as determined by Sytox Green, but might moderately alter the physical integrity of the outer membrane structure through its interaction with lipid A. The potential use of this fragment as antimicrobial is promising, as it additively enhanced the killing of bacteria when used with several conventional antibiotics. Its bactericidal activity could be improved by genetically modifying its structural amphipathicity. Furthermore, since rfhSP-A recognizes Gram-negative bacteria, the possibility of using this fragment as carrier for small antimicrobial molecules that bind to rfhSP-A could help develop new alternative treatments against Gram-negative bacteria.

## Data availability statement

The raw data supporting the conclusions of this article will be made available by the authors, without undue reservation.

## Author contributions

Conceptualization, JC, VF-Á, LT, AS, BG-F, JB, NK, JJ, and CC; Methodology and Investigation, JC, VF-Á, LT, AS, BG-F, and NK. Contribution with tools and expertise, JB, and JJ. Formal analysis, all authors. Writing—original draft preparation, JC and VF-Á; Writing—review and editing, CC; Supervision, CC; Project administration, CC Funding acquisition for this study, JB, JJ, and CC. All authors read and agreed to the published version of the manuscript.

## Funding

This study was supported by the Spanish Ministry of Science, Innovation and Universities through Grants SAF2015-65307-R and RTI2018-094355‐B‐I00 to CC, and by the Swedish Research Council (2020-02434) to JJ, and by the Biotechnology and Biological Sciences Research Council (BB/T001976/1 and BB/L007223/1) and Medical Research Council (MR/R005893/1) to JB. VF-Á was the recipient of a postdoctoral contract from Complutense University of Madrid (POP-UCM, 2021-2022, CT17/17-CT18/17) and LT was the recipient of a contract (PEJ-2020-AI/BMD-17865) from the Consejería de Educación, Juventud y Deporte of Comunidad de Madrid (Spain) and the European Social Funding Program.

## Acknowledgments

We thank the Confocal Microscopy Unit of the Complutense University of Madrid for their excellent technical support. We also thank Dr. Junkal Garmendia (Instituto de Agrobiotecnología, Mutilva, Spain) for providing nontypeable *H. influenzae* strains used in this study.

## Conflict of interest

The authors declare that the research was conducted in the absence of any commercial or financial relationships that could be construed as a potential conflict of interest.

## Publisher’s note

All claims expressed in this article are solely those of the authors and do not necessarily represent those of their affiliated organizations, or those of the publisher, the editors and the reviewers. Any product that may be evaluated in this article, or claim that may be made by its manufacturer, is not guaranteed or endorsed by the publisher.
