# Supplementary material for: Cooperative action of SP-A and its trimeric recombinant fragment with polymyxins against Gram-negative respiratory bacteria
Source: Front Immunol. 2022 Sep 7;13:927017. doi: 10.3389/fimmu.2022.927017 (PMC9493720; doi:10.3389/fimmu.2022.927017)
Supplement: Supplementary file 1 [file DataSheet_1.pdf]

Supplementary Material

Cooperative action of SP-A and its trimeric recombinant fragment with polymyxins against Gram-negative respiratory bacteria

Juan Manuel Coya[1,†], Víctor Fraile-Ágreda[1,†], Lidia de Tapia[1], Belén García-Fojeda[1], Alejandra Sáenz[1], , José A. Bengoechea[2], Nina Kronqvist[3], Jan Johansson[3] , and Cristina Casals[1*].

[1] Department of Biochemistry and Molecular Biology, Complutense University of Madrid, Madrid, Spain.

[2] Wellcome-Wolfson Institute for Experimental Medicine, Queen's university Belfast, Belfast, UK.

[3]Department of Biosciences and Nutrition, Neo, Karolinska Institutet, Huddinge, Sweden.

(†) These authors have contributed equally to this work.

***Correspondence:**
Cristina Casals.
Department of Biochemistry and Molecular Biology, Faculty of Biology, Complutense University of Madrid, 28040 Madrid, Spain,
Tel.: (34) 913944261,
ccasalsc@ucm.es

Keywords: Collectin SP-A, recombinant trimeric fragment**,** multidrug-resistant bacteria, microbial infection, lung, polymyxin B, PMB nonapeptide, synergy

Running Title**: Synergy of SP-A and polymyxins**

Number of words: 8500
Number of Figures: **13 and 1 Table (3 Supplementary Figures)**

Research Topic in Molecular Innate Immunity: Updates on the Role of Surfactant Proteins A and D in Innate Immune Responses

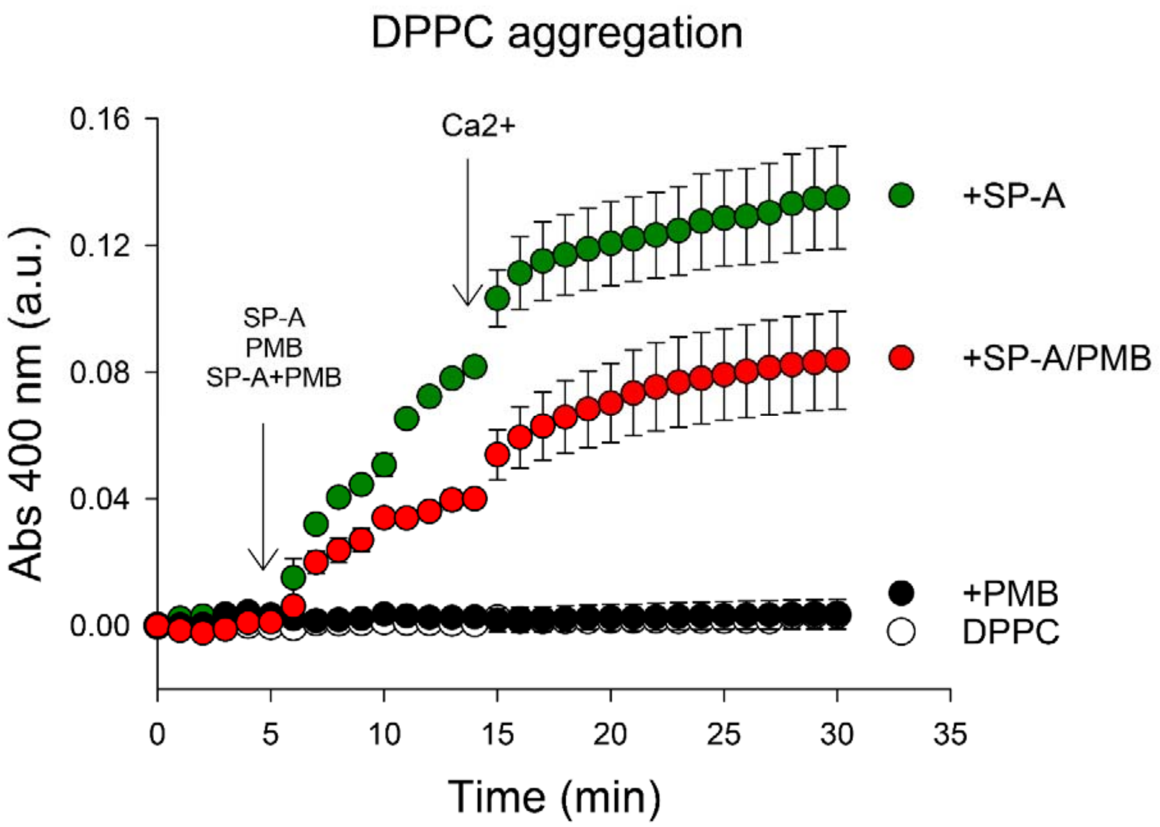

Supplementary Figure 1. DPPC vesicle aggregation induced by SP-A in the presence and absence of PMB. Sample and reference cuvettes were filled with 50 µg/ml of DPPC vesicles in 5 mM Tris/HCl buffer, pH 7.4, containing 150 mM NaCl, and 1 mM EDTA. After 10 min equilibration at 37 °C, either SP-A, PMB or PMB+SP-A (final concentration 50 µg/ml) was added to the sample cuvette. Next, 2.5 mM Ca^{2+} was added to the sample and cuvettes.

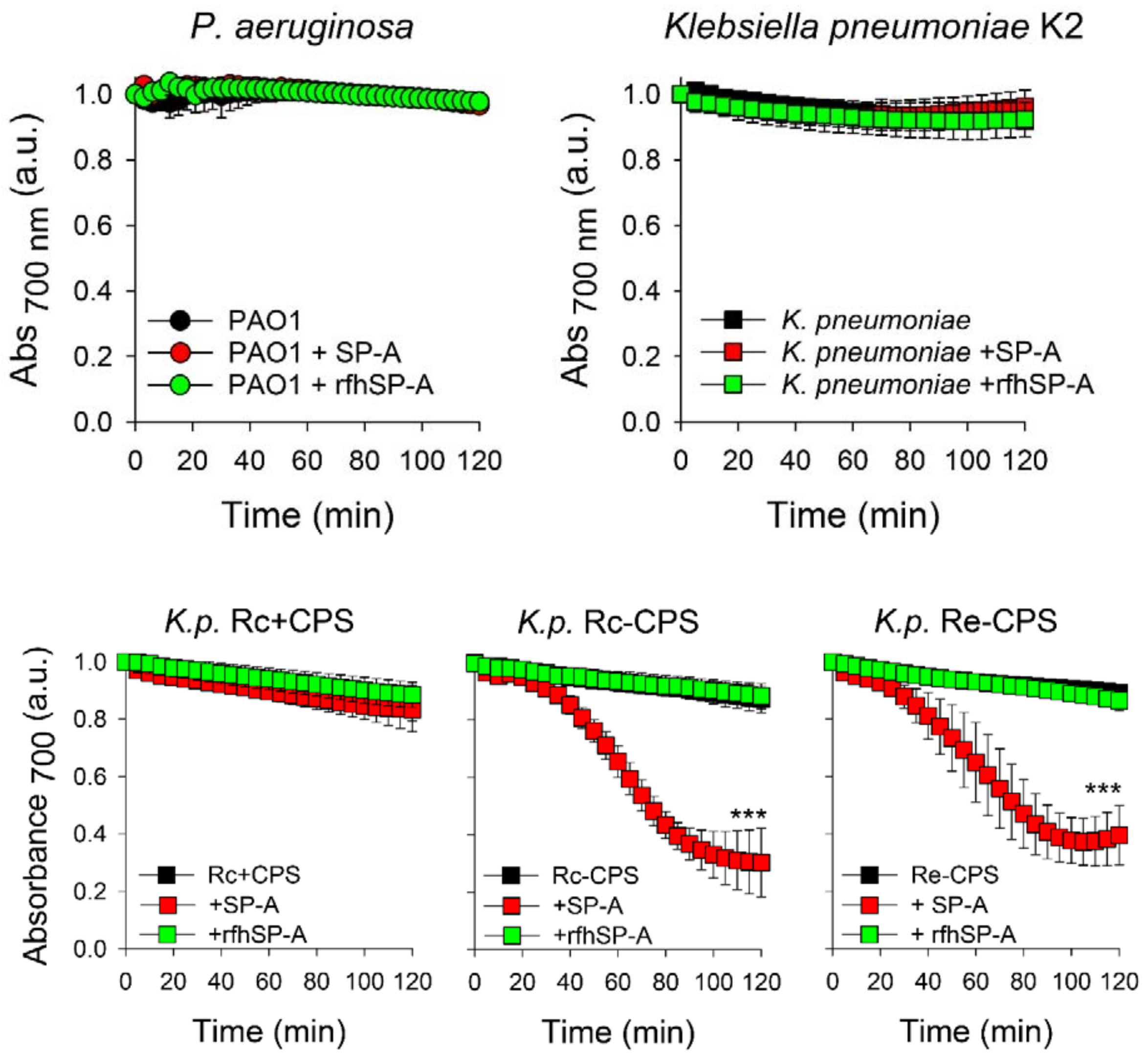

Supplementary Figure 2. Aggregation of *P. aeruginosa* and *K. pneumoniae* and isogenic mutants Re and Rs (with and without capsule) of *K. pneumoniae* in the absence and presence of SP-A (25 μg/ml) and rfhSP-A (12.5 μg/ml). Bacterial aggregation is observed as a decrease in absorbance at 700 nm during 120 min as bacterial aggregates precipitate out of solution.

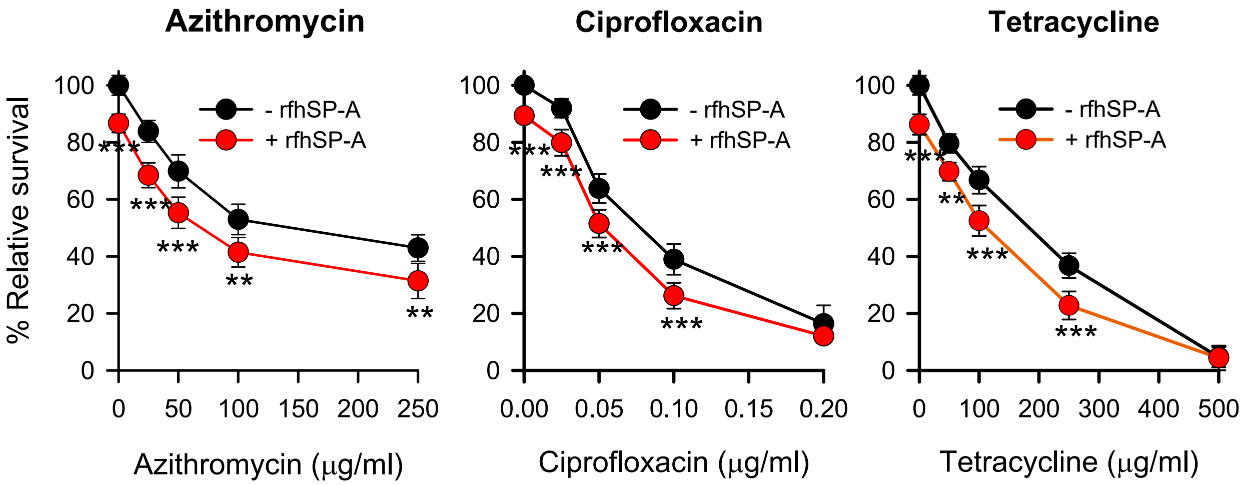

Supplementary Figure 3. rfhSP-A in combination with antibiotics increases the killing of *K. pneumoniae*. 10^5 CFUs/ml of bacteria were incubated with different concentrations of antibiotics in the absence or presence of rfhSP-A (1 μg/ml) (17.5 nM) in 10 mM phosphate, 1% TSB, and 100 mM NaCl buffer (pH 7.4) for 1 h at 37 °C. Bacteria were then plated on LB agar for CFU counting. Results are shown as a percentage of relative survival compared to untreated bacteria. Data are means ± SD of three independent experiments, each in triplicate. Results were statistically analyzed by Student's t-test. *p < 0.05, **p < 0.01, and ***p < 0.001 when bacteria treated with rfhSP-A + antibiotic were compared with bacteria treated with the antibiotic alone.
